# Polyamine Pathway Inhibitor DENSPM Suppresses Lipid Metabolism in Pheochromocytoma Cell Line

**DOI:** 10.3390/ijms251810029

**Published:** 2024-09-18

**Authors:** Hans K. Ghayee, Kaylie A. Costa, Yiling Xu, Heather M. Hatch, Mateo Rodriguez, Shelby C. Straight, Marian Bustamante, Fahong Yu, Fatima Smagulova, John A. Bowden, Sergei G. Tevosian

**Affiliations:** 1 Department of Medicine, Division of Endocrinology, College of Medicine, University of Florida and Malcom Randall VA Medical Center, Gainesville, FL 32608, USA; yiling.xu@ufl.edu (Y.X.); marianbustamante@ufl.edu (M.B.); 2 Department of Physiological Sciences, College of Veterinary Medicine, University of Florida, Gainesville, FL 03610, USA; kcosta@ufl.edu (K.A.C.); maeve@ufl.edu (H.M.H.); mrodriguezz@ufl.edu (M.R.); sstraight@ufl.edu (S.C.S.); john.bowden@ufl.edu (J.A.B.); 3 The Interdisciplinary Center for Biotechnology Research, University of Florida, Gainesville, FL 32610, USA; fyu@ufl.edu; 4 Université de Rennes, EHESP, Inserm, Irset (Institut de Recherche en Santé, Environnement et Travail), Campus Sante de Villejean—UMR_S 1085, F-35000 Rennes, France; fatima.smagulova@inserm.fr

**Keywords:** pheochromocytoma, paraganglioma, SDHB, DENSPM, plasmanyl

## Abstract

Pheochromocytomas (PCCs) are tumors arising from chromaffin cells in the adrenal medulla, and paragangliomas (PGLs) are tumors derived from extra-adrenal sympathetic or parasympathetic paraganglia; these tumors are collectively referred to as PPGL cancer. Treatment for PPGL primarily involves surgical removal of the tumor, and only limited options are available for treatment of the disease once it becomes metastatic. Human carriers of the heterozygous mutations in the succinate dehydrogenase subunit B (*SDHB*) gene are susceptible to the development of PPGL. A physiologically relevant PCC patient-derived cell line hPheo1 was developed, and *SDHB*_KD cells carrying a stable short hairpin knockdown of *SDHB* were derived from it. An untargeted metabolomic approach uncovered an overactive polyamine pathway in the *SDHB*_KD cells that was subsequently fully validated in a large set of human *SDHB*-mutant PPGL tumor samples. We previously reported that treatment with the polyamine metabolism inhibitor N^1^,N^11^-diethylnorspermine (DENSPM) drastically inhibited growth of these PCC-derived cells in culture as well as in xenograft mouse models. Here we explored the mechanisms underlying DENSPM action in hPheo1 and *SDHB*_KD cells. Specifically, by performing an RNAseq analysis, we have identified gene expression changes associated with DENSPM treatment that broadly interfere with all aspects of lipid metabolism, including fatty acid (FA) synthesis, desaturation, and import/uptake. Furthermore, by performing an untargeted lipidomic liquid chromatography–mass spectrometry (LC/MS)-based analysis we uncovered specific groups of lipids that are dramatically reduced as a result of DENSPM treatment. Specifically, the bulk of plasmanyl ether lipid species that have been recently reported as the major determinants of cancer cell fate are notably decreased. In summary, this work suggests an intersection between active polyamine and lipid pathways in PCC cells.

## 1. Introduction

Pheochromocytomas (PCCs) are tumors that arise from the chromaffin cells in the adrenal medulla. Extra-adrenal PCCs are known as paragangliomas (PGLs) and arise from the sympathetic and parasympathetic chain ganglia [1]. Collectively these tumors are referred to as PPGL. The mainstay for therapy in the majority of PPGL patients is surgical resection of the tumor with adequate alpha adrenoceptor blockade prior to surgery [2]. The World Health Organization (WHO) identified all PPGL cancers as having potential for metastatic behavior [3]. Recent data have provided new insights in improved understanding of these potentially lethal tumors. Specifically, advances in genomics have uncovered over 20 different driver mutations that may lead to PPGL [4,5]. Germline and somatic pathogenic variants encoding proteins comprising a tricarboxylic acid (TCA)/Krebs cycle enzyme succinate dehydrogenase *SDHx* are considered to be one of the most important in the pathogenesis of these tumors. The Succinate Dehydrogenase Complex (SDHx) resides in the inner membrane of the mitochondria and is essential to both the tricarboxylic acid cycle and the oxidative phosphorylation chain. Human carriers of the heterozygous mutations in the succinate dehydrogenase subunit B (*SDHB*) are susceptible to the development of oncological diseases with poor prognosis. Currently there is no cure for patients with metastatic PPGL. 

Our laboratory recently performed a metabolomic analysis of *SDHB*_KD, *SDHB*-mutant human progenitor cells developed from hPheo1, a physiologically relevant PCC patient-derived cell line [6] and discovered an overactive polyamine pathway subsequently fully validated in human PPGL *SDHB*-mutant tumor samples [7]. Polyamines (putrescine, spermidine, and spermine) are polycations that have been ascribed key roles in multiple cellular processes: modulation of chromatin structure, gene transcription and translation, DNA stabilization, signal transduction, cell growth, proliferation and migration, membrane stability and ferroptosis, and functioning of ion channels and receptor-ligand interactions. In mammalian cells, putrescine is produced through the decarboxylation of ornithine (that is derived from arginine through the action of arginase, ARG) by ornithine decarboxylase (ODC). ODC is the rate-limiting enzyme in the polyamine biosynthesis pathway and is subjected to multiple levels of regulation. Cellular polyamine levels are carefully calibrated due to their critical role in supporting cell proliferation and their potential toxicity at excessive levels. This regulation occurs at four levels: de novo synthesis, interconversion, catabolism, and transport/uptake. Depleting intracellular polyamine pools leads to a block in the cell cycle and suppression of cell growth; specifically, inhibiting ODC with its specific inhibitor difluoromethylornithine (DFMO) causes a G1-phase arrest [8]. Most pertinently, polyamine analogue inhibitors (such as AMXT 1501, DEHSPM, and DENSPM) resembling naturally occurring molecules have been designed that interfere both with the transport/uptake and synthesis of polyamines [9]. 

Elevated polyamine levels are usually associated with increased cell proliferation, reduced apoptosis, and enhanced expression of genes linked to tumor invasion and metastasis, making their metabolism a potential target for cancer treatment and prevention. Several recent publications illuminate the central role of polyamines in neoplastic diseases (reviewed in [10]), including tumors of neuronal origin. For example, co-administration of AMXT 1501 and DFMO leads to in vitro suppression of growth and significant extension of survival in three aggressive diffuse intrinsic pontine gliomas (DIPG) orthotopic animal models, demonstrating the potential of dual targeting of polyamine synthesis and uptake as a promising therapeutic strategy for incurable DIPG [11]. Gamble et al. demonstrated that the MYC-N proto-oncogene modulates polyamine catabolism, synthesis, and transport in neuroblastomas (NBs). Here, similarly, inhibition of polyamine uptake with AMXT-1501 in combination with DFMO effectively treated MYCN-amplified NBs in transgenic and PDX mouse models [12]. Recent research also uncovered the pro-ferroptotic function for polyamines and the corresponding positive-feedback loop whereby polyamine catabolism amplifies ferroptosis [13]. In summary, metabolism and transport of polyamines in neoplastic cells could be successfully targeted in cancer treatment [7,10,14]; however, the mechanisms of polyamine action in PPGL remain to be understood. 

Previously, we demonstrated that treatment with the polyamine metabolism inhibitor N(1),N(11)-diethylnorspermine (DENSPM) drastically inhibited growth of a *SDHB*-knockdown line (*SDHB*_KD) we have developed [7], and DENSPM was effective against tumors derived from both hPheo1 as well as *SDHB*_KD cells in a xenograft mouse model [7]. Despite notable suppression of tumor cells growth, the specific pathway downstream of DENSPM action in PCC-derived cells remained unclear. Here we explored the mechanism underlying this polyamine analogue drug action in hPheo1 cells. Specifically, by performing an RNAseq analysis in hPheo1 cell line and its *SDHB*_KD derivate we have identified biochemical processes associated with DENSPM treatment that broadly interfere with all aspects of lipid metabolism, including fatty acid (FA) synthesis, desaturation, and import/uptake. Furthermore, by performing an untargeted lipidomic liquid chromatography–mass spectrometry (LC/MS)-based analysis we uncovered specific groups of lipids that are dramatically reduced as a result of DENSPM treatment.

## 2. Results

### 2.1. RNAseq Analysis Reveals DENSPM’s Effect on Fatty Acid Metabolism Gene Expression

**Figure 1:**
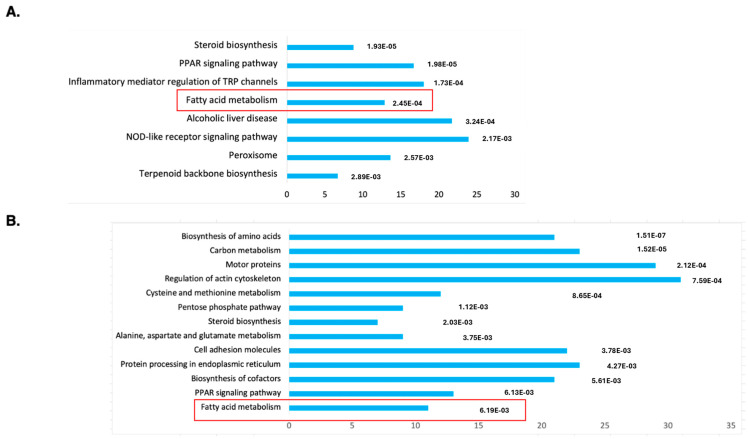
Functional annotation as “biological process”, “cellular localization” or “molecular function” of genes identified as downregulated by the RNA-seq analysis of DENSPM treated hPheo1 (**A**) or SDHB_KD (**B**) cells. Bars were sorted by adjusted *p*-values; the length of each bar represents the number of genes in each group. The fatty acid metabolism category is indicated by a red frame.

To understand the molecular mechanism of DENSPM action in hPheo1 cells, we performed an RNAseq analysis of gene expression to compare untreated and treated cells. Principal Component Analysis (PCA) demonstrated a complete separation of the treated vs. untreated group (Supplementary Figure S1). The gene list was generated with 2168 downregulated/2827 upregulated genes, and differentially expressed genes were further analyzed against The Database for Annotation, Visualization and Integrated Discovery (DAVID; https://david.ncifcrf.gov; accessed on 11 January 2024) using KEGG pathway analysis (Figure 1A). The gene set was also examined using Gene Set Enrichment Analysis (GSEA, https://www.gsea-msigdb.org/gsea/index.jsp; accessed on 16 January 2024), the Molecular Signatures Database (MSigDB) (Supplementary Figure S2A) and Ingenuity Pathway Studio (Supplementary Figure S3). We noted that the “fatty acid metabolism” gene set is among only three enriched sets (out of total 40) with *p*-value below <0.01 determined by GSEA (Supplementary Figure S1A). It was also the only gene set identified by both the DAVID and GSEA approaches. 

**Figure 2:**
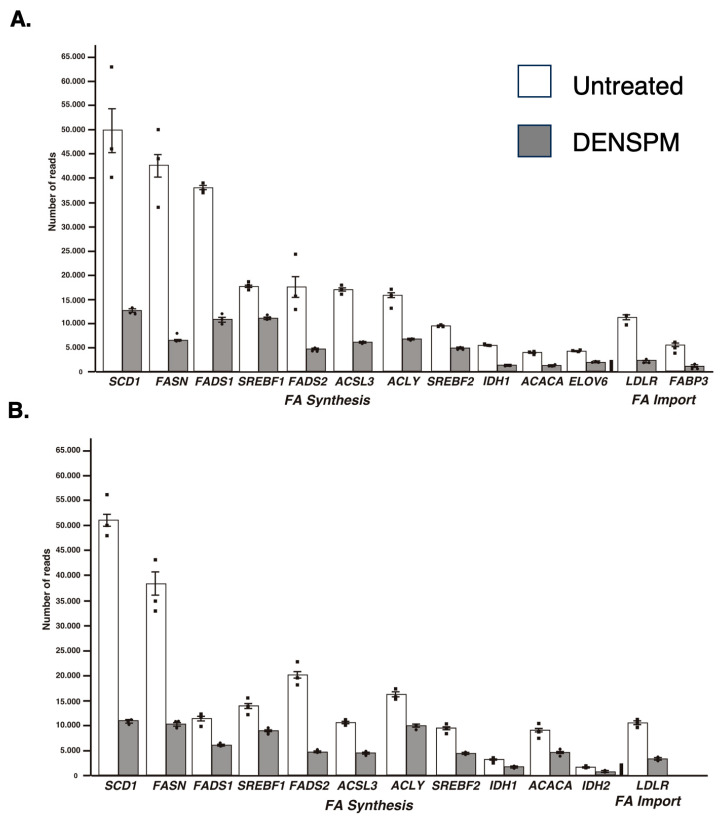
Relative gene expression of lipid-associated genes from the RNA-Seq analysis of hPheo1 (**A**) and SDHB_KD (**B**) cells. The means and SEMs for the number of reads corresponding to either untreated (white bars) or DENSPM-treated (grey bars) cells are shown; black squares represent individual observation. All sets are highly significant (*p* < 0.001).

While the Ingenuity Pathway Analysis did not list the fatty acid metabolism pathway, an overlapping set of genes was uncovered in categories of “Super-pathway of Cholesterol Biosynthesis” and “Cholesterol Biosynthesis I, II and III pathways” (Supplementary Figure S3). It also detected “Lipid Metabolism” as the top category under the umbrella of the “Molecular and Cellular Functions” pathways. Similarly, “Fatty acid metabolism, Synthesis of lipid” was identified as the “Top Regulator Effect Network”. We further examined the RNAseq data for a comprehensive set of genes involved in lipid metabolism (Figure 2A). This set was compiled directly from genes flagged by the computer programs and by a manual search for fatty-acid-metabolism-associated gene tags based on analysis of the literature (e.g., [15,16]). These include genes associated with de novo lipid synthesis as well as lipid import/uptake. As shown in Figure 2A, all genes in the lipid-associated pathway were downregulated upon DENSPM treatment in hPheo1 cells. We also conducted the analysis of the cells carrying a shRNA that interferes with *SDHB* gene expression, *SDHB*_KD [7]. Similarly, “fatty acid metabolism” was identified by the gene expression analysis programs (Figure 1B) and the same gene set was found to be downregulated in these cells (Figure 2B) In summary, RNAseq analysis of the hPheo1 and *SDHB*_KD transcriptomes indicated that DENSPM treatment comprehensively downregulates expression of lipid-metabolism-associated genes.

### 2.2. RT-PCR Analysis of Gene Expression

**Figure 3:**
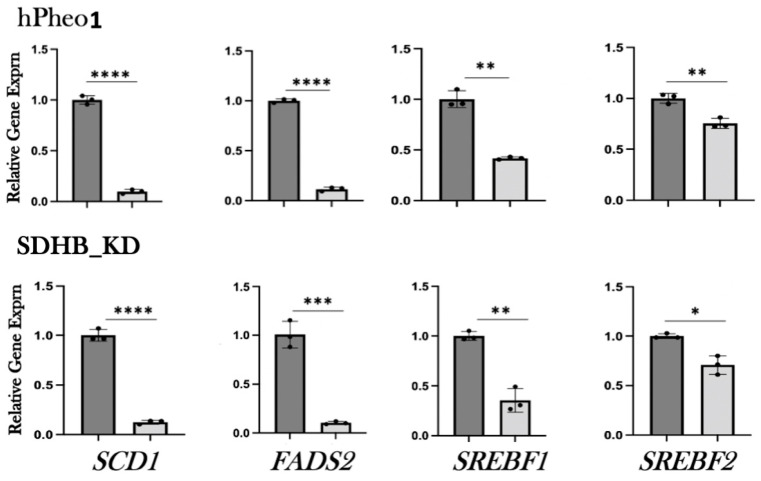
Relative gene expression of select lipid-associated genes identified by RNA-seq was confirmed by qRT-PCR. Relative gene expression corresponding to either untreated (dark gray bars) or DENSPM-treated (light grey bars) cells is shown. The data are presented as means and SEMs. ****, *p* < 0.0001; ***, *p* < 0.001; **, *p* < 0.01 *, *p* < 0.05.

To confirm the results of the RNAseq analysis, we tested the expression of the subset of genes shown in Figure 2 by qRT-PCR. Both hPheo1 and *SDHB*_KD cells were included in this analysis. To this end, RNA was extracted, cDNA was synthesized, and qPCR was performed. We observed downregulation of lipid-associated gene expression upon DENSPM exposure of all the genes we tested in both cell types (Figure 3 and Table 1). We also confirmed downregulation of an additional gene set associated with lipid metabolism using the RNA from the hPheo1 cells (Supplementary Figure S4 and Supplementary Table S1). In summary, qRT-PCR analysis of the gene expression validated the RNAseq analysis of the changes in the transcriptomes of PCC-derived cells upon DENSPM treatment.

### 2.3. Western Blotting Analysis

**Figure 4:**
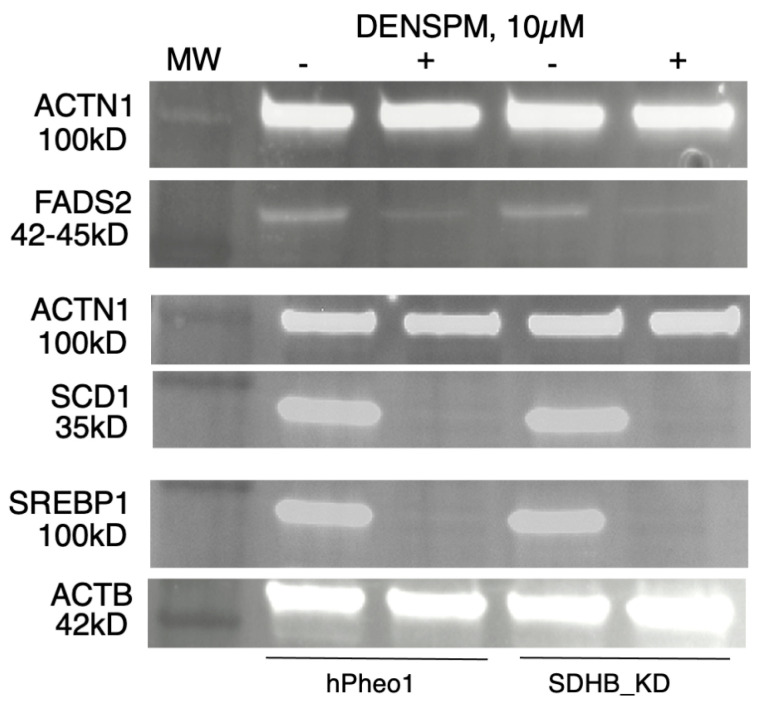
Western blot analysis of FADS2, SCD, and SREBP1 protein expression in hPheo1 (left) and SDHB_KD (right) cells untreated (−) or treated (+) with 10 µM DENSPM.

To examine gene expression at the protein level we performed a Western Blotting analysis of several proteins (SCD1, FADS2, and SREBP1) corresponding to the genes in Figure 1, Figure 2 and Figure 3. WB analysis showed near absence of these proteins in both types of cells upon DENSPM treatment (Figure 4). This confirms that both RNA as well as proteins involved in lipid metabolism are suppressed in PCC-derived cells upon DENSPM treatment.

### 2.4. Bioinformatics Analysis of Gene Expression in PPGL Tumors

**Figure 5:**
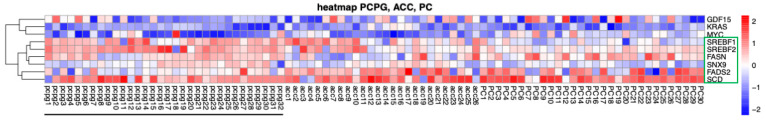
Heatmap of the 9 genes (including 6 genes associated with lipid metabolism, green box) that are differentially expressed between PPGL (underlined), ACC, and Prostate Cancer (PC) tumors. Transcript names are shown along the right axis. Red: increased expression, blue: decreased expression.

To explore the expression of fat-metabolism-associated genes in PPGL tumors we performed a bioinformatics analysis. RNA seq profiles were uploaded from the TCGA portal (https://portal.gdc.cancer.gov/projects/accessed on 23 April 2024) using 32 cases of RNA-seq data from pheochromocytomas and paragangliomas (PPGLs), 26 for adrenocortical carcinoma (AC) and 30 for prostate cancer (PC). Transcript per million counts were extracted for each gene of interest from the RNAseq data. TPM values were Log2 transformed to reduce variations between highly expressed genes and low-expressed genes. A heatmap was built using R script using the Pheatmap package. In addition to the PPGL tumors, we included in this comparison adrenocortical carcinoma (ACC) tumors as these were shown to rely on lipid metabolism [22,23,24,25]. We determined that expression of several genes associated with lipid metabolism is notably upregulated in the ACC and PPGL tumors compared to other genes (e.g., MYC), but not in the PC tumors (Figure 5).

### 2.5. Lipidomics Profiling in hPheo1 and hPheo1-SDHB_KD Cells

**Figure 6:**
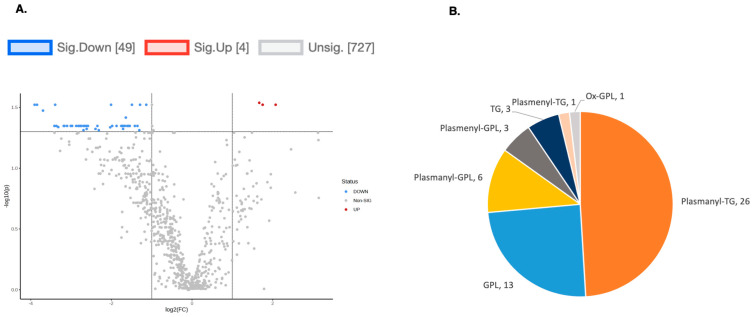
(**A**) A volcano plot of the LC/MS analysis of lipid composition in untreated vs. 10 µM DENSPM-treated hPheo1 Cells (fold change = 2.0; *p*-value = 0.05, FDR adjusted). (**B**) A Pie chart shows the significantly altered lipids by (sub)class. Note that the majority of the lipids belong to the plasmanyl class.

**Figure 7:**
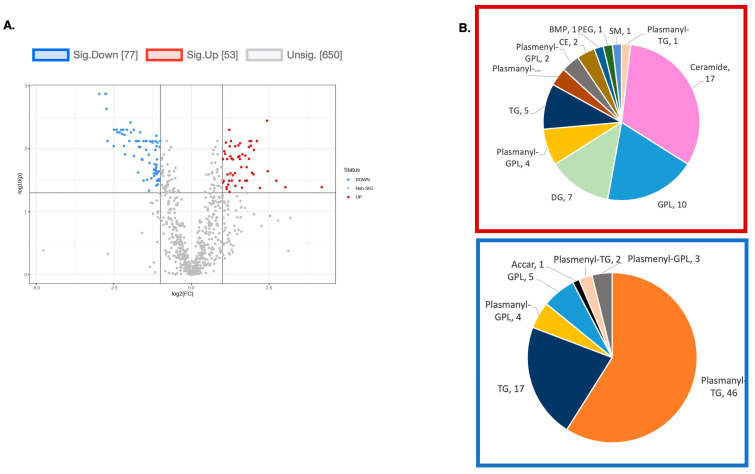
(**A**) A volcano plot of the LC/MS analysis of lipid composition in untreated vs. 10 µM DENSPM-treated *SDHB*_KD Cells (Fold change = 2.0; *p*-value = 0.05, FDR adjusted). (**B**) A Pie chart shows the significantly altered lipids by (sub)class. Red box: significantly increased lipids by (sub)class. Blue box: significantly decreased lipids by (sub)class. Note that the majority of downregulated lipids belong to the plasmanyl class.

**Figure 8:**
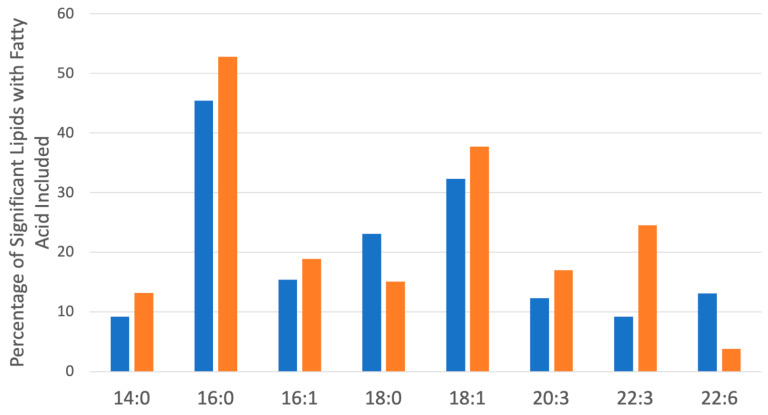
Most common saturated and unsaturated fatty acids within the significantly changed lipids (Figure 6 and Figure 7) in hPheo1 (Orange) and SDHB_KD cells (Blue) treated with DENSPM. Fatty acid classes comprising over 10% of the significant lipids are included. No significant changes between hPheo1 and SDHB_KD cells were noted.

To explore the functional consequences of a comprehensive decline in the expression of lipid-related genes and processing enzymes in hPheo1 and *SDHB*_KD cells upon DENSPM treatment, we performed lipidomics profiling using an LC/MS approach. LS/MS analysis identified 53 (49 decreased, 4 increased) lipids that changed significantly (Fold Change = 2; *p* = 0.05, with FDR correction) upon DENSPM treatment in hPheo1 cells (Figure 6A). Almost half of these significantly changed lipids belonged to the class of ether plasmanyl lipids (Figure 6B). Specifically, plasmanyl-triglycerides constitute the largest share, with 31% (26 out of 83) of the lipids in this class significantly decreased. In the *SDHB*_KD cells we identified approximately twice as many (130) lipids that changed significantly upon treatment. Of these 130 significantly changed lipids, 77 were decreased and 53 were increased in the treatment versus control *SDHB*_KD cells (Figure 7A). Similarly, the most significantly downregulated lipids (58%, 48 out of 83) belonged to the plasmanyl-triglyceride group (Figure 6B). In the “significantly increased” group of lipids for *SDHB*_KD cells (which was notably larger than for hPheo1, 53 vs. 4), most species belong to ceramides or glycerophospholipids class (Figure 7B). Examination of common fatty acid compositions of the significantly changed lipids in both the hPheo1 cells and *SDHB*_KD cells demonstrated that they are enriched in specific fatty acids; namely, 16:0, 16:1, 18:0, and 18:1 fatty acids were highly prevalent within the significant lipids (Figure 8). No significant differences between the hPheo1 and *SDHB*_KD cells were noted in acid composition. The impact of these changes in fatty acid composition is currently under investigation.

## 3. Discussion

The involvement of fatty acids (FA) and lipids in cancer recently became an area of active research. Lipids are a complex group of biomolecules with diverse cellular functions such as membrane organization, permeability and integrity, energy storage, and signaling. The pathogenesis of lipid defects is associated with several diseases, including cancer. In cancer, lipids are implicated in supporting metastasis, and high-fat diets promote tumor spread by creating pre-metastatic niches in mice [26,27,28], while low-fat diets suppress tumor growth [29,30]. FAs play a crucial role in serving as an energy source and building biological membranes, which are highly demanded in rapidly dividing neoplastic cells. Additionally, FAs contribute to generating lipid signaling molecules like phosphoinositides, sphingolipids, and eico- and doco-sanoids. Specific fatty acids are also vital for the acylation of cancer-related proteins such as Wnt and Ras [31,32]. Another important role of FAs is the promotion of lipid accumulation in lipid droplets (LDs), which serve as storage sites for excess fatty acids and cholesterol. When tumors grow and exogenous nutrients become scarce, cancer cells can consume fatty acids released from LDs through lipolysis to fulfill their energy requirements. LDs are also utilized to lock out damaged or highly oxidized lipids and unfolded proteins, providing protection against lipotoxicity, lipid peroxidation, and endoplasmic reticulum (ER) stress [33]. Cancer stem cells (CSCs) exhibit distinct metabolic features compared to non-CSCs, one of which is altered lipid metabolism, paving the way for the identification of unique vulnerabilities that can be exploited for targeting this cell population (reviewed in [34]). In summary, FAs are suspected to carry several roles in cancer development, progression, and subsequent metabolic rewiring; however, tumor-specific pathways downstream of FAs for most cancers, including PPGL, are not known [35].

Several lines of evidence point to dysregulation of lipid metabolism as a key part of mitochondrial dysfunction, specifically the one driven by the *SDHB* pathogenic variants. Specifically, individuals with *SDHx* deficiency show changes in serum indicating a shift in their FA metabolism, specifically an increase in the elongation of saturated FAs and a higher level of desaturation of FAs in patients with *SDHC/D* variants. The authors also observed a rise in the C20–C24 FAs in patients who have PPGL in combination with *SDHx* genetic deficiency [36].

Moreover, in a mouse model of *SDHB* loss (pancreatic ß cells deficient in *SDHB* (*SDHB*^ßKO^)), a combination of transcriptomic and metabolomic analysis identified fatty acid, lipid, and cholesterol metabolism pathways, including SREBP-regulated cholesterol and fatty acid biosynthesis as the most significantly altered in *SDHB*^βKO^ islets [37]. Another noted intersection is between the SDHx-dependent pathway and polyamine metabolism lies in the regulation of T-cell differentiation. The SDHx complex is critical to T-cell regulation and its inhibition leads to a drastic impairment of T-cell proliferation and cytokine secretion. These changes represent an integral part of T-cell functionality and induce a proinflammatory gene signature in T cells, promoting T-helper (T_H_) lineage differentiation [38,39]. Similarly, recent research shows that the polyamine pathway is key to guiding CD4+ helper T_H_ differentiation and function [37,40,41,42]. The exact mechanisms underlying these *SDHB*-related alterations in metabolism are not yet fully understood, and further research was needed to clarify the relationship between the polyamine pathway, lipid metabolism, and PPGL.

Now, we demonstrated that treatment of PCC-cancer-derived cell lines with a polyamine analogue inhibitor DENSPM results in a broad downregulation of gene expression associated with fatty acid synthesis and processing, including *FASN*, *SCD1*, *FADS2*, and *SREBPs*. De novo synthesis of FAs in adult tissues is mostly limited to the liver, adipose tissue, and lactating breasts [43]. However, it has been known for a long time that tumor cells are able to convert glucose or acetate into lipids at a rate similar to that observed in the liver [44,45]. An activated lipid production that enables versatile tumor cells to synthesize, elongate, and desaturate fatty acids and support proliferation and membrane biogenesis has been documented in many cancers (e.g., [15,16,46,47,48]). A particular subset of tumor cells is specifically sensitive toward approaches targeting fatty acid metabolism, in particular fatty acid desaturation [49,50,51,52]. Specifically, fatty acid synthase (FASN), the rate-limiting enzyme in the de novo FA synthesis pathway has been widely reported to act as a pro-oncogenic enzyme and promote cancer progression. FASN is upregulated in several types of cancers and it is critical for boosting FA production. FASN supports cell proliferation through augmenting membrane biosynthesis and promoting invasion, metastasis, and angiogenesis by facilitating the formation of lipid rafts [43,47,53,54].

FAs synthesized de novo through FASN action are fully saturated; hence, a substantial fraction of de novo synthesized FAs will require desaturation by the activity of stearoyl-CoA desaturase (SCD), a critical modulator of the fatty acid metabolic pathway [55]. Similar to FASN, SCD has been strongly implicated in the development and progression of neoplastic disease including a neuroblastoma [28,56,57,58,59,60,61,62] that is also vulnerable to treatment with polyamine pathway inhibitors. High *SCD1* expression is associated with poor prognosis in several cancer types. Recently, it was shown that elevated levels of SCD protect tumor cells against programmed cell death and ferroptosis (see also below) [63]. Pharmacological inhibition of SCD showed promising anti-tumor potential in preclinical models [28]. A recent publication underscored critical importance of desaturation for cancer cells that exploits a metabolic rewiring process and engages an alternative fatty acid desaturation pathway using the fatty acid desaturase 2 (FADS2) enzyme instead of the conventional desaturation by SCD [50]. In these cells FADS2 desaturates the FA palmitate into the unusual the FA sapienate, which supports tumor cells’ membrane biosynthesis during proliferation. This demonstrates the need to inhibit both desaturation pathways to impair the in vitro and in vivo proliferation of cancer cells that can synthesize sapienate. *FADS2* expression is prognostic in some cancers, and FADS2-mediated sapienate metabolism is regulated by mTOR signaling [64]. Importantly, PPGL tumors demonstrate a high level of expression for *FASN*, *SCD1*, and *FADS2* ([64] and Figure 5). Hence, the ability of DENSPM to simultaneously suppress FASN, SCD, and FADS2 could be of critical importance for successful tumor treatment.

While de novo FA synthesis is the prevailing route of lipid acquisition in cancer cells, studies have shown that tumors can also obtain exogenous FAs by upregulating various FA-uptake mechanisms. Oxygen and nutrient deprivation in the tumor often limit its metabolic flexibility to switch between different substrates for intrinsic FA production. To overcome that, cancer cells will attempt to increase FA uptake to compensate for reduced glucose-based de novo FA synthesis. Hence, therapeutic strategies simultaneously targeting several routes of lipid provision, including import, could be advantageous depending on cell type [65,66]. Hypoxia increases FA transport in breast, ovarian and glioblastoma cancer cells by inducing the expression of FA-binding proteins (FABP3, FABP7, or FABP4), which are involved in the uptake and subcellular trafficking of FAs [67,68,69]. High expression of LDLR in several cancers is associated with poor prognosis and reduced survival, indicating that LDLR is an independent adverse predictive marker [70,71,72] and targeting LDLR expression showed a promising effect as a cancer therapy [73,74]. Similarly, targeting the CD36 translocase in metastasis-initiating cells by neutralizing antibodies causes almost complete inhibition of metastasis in immunodeficient or immunocompetent orthotopic mouse models of human oral cancer [75]. While gene knockdowns/knockouts and inhibitors for most of the proteins intimately involved in lipid metabolism and transport have been described, achieving comprehensive suppression of lipid metabolism proved to be far from trivial [76]. In this respect, it is important that the polyamine pathway inhibitor DENSPM broadly targets the ability of PPGL cancer cells to both synthesize and acquire lipids.

Perhaps, the most tantalizing outcome of DENSPM treatment is a dramatic reduction in ether lipids (Figure 6 and Figure 7). Increased levels of ether lipids in cancer cells were reported over half a century ago [77]. Recent work demonstrated that alkylglyceronephosphate synthase (AGPS), a key enzyme in the generation of ether lipids, is overexpressed in aggressive human cancer cells and primary tumors. AGPS inactivation by hairpin RNAs was shown to impair cancer cells survival, motility, invasiveness, and anchorage-independent growth, as well as their potency to produce xenograft tumors in an animal model. On the contrary, AGPS overexpression increased cell tumorgenicity [78].

Despite these important studies showing the correlation between ether lipid levels and cancer cell potency, a mechanistic understanding of ether lipids’ role in cancer development was lacking. Our knowledge of the role for the ether lipids has been recently transformed. Henry et al. reported that ether lipids are key determinates of the biophysical characteristics for membranes in cancer cells with high metastatic potential, where they act to keep membrane tension low and membrane fluidity high [79]. One of the key findings in this work concerns the contribution of ether phospholipids to ferroptosis. Previously it was believed that unsaturated fatty acids in ether lipids simply serve as substrates for iron-catalyzed peroxidation. Henry et al. now prove that ether lipids’ major role is in modifying membrane permeability and directly promoting iron import. Through a combination of sophisticated genetic approaches and lipid reconstitution tests, the authors demonstrated that these biophysical properties regulated by ether lipids allow for non-clathrin-mediated iron endocytosis through the CD44 pathway, resulting in a substantial increase in intracellular redox-active iron and conferring a higher susceptibility to ferroptosis. The authors further showed that in the absence of ether lipids key characteristics of cancer cells such as extravasation, metastatic load, and cancer stemness are significantly reduced. The findings of Weinberg and colleagues expose a key duality whereby ether lipids act in carcinoma cells as crucial drivers of malignant progression while at the same time also offer a vulnerability point that can be targeted for therapeutic intervention.

Our lipidomic analysis demonstrated that DENSPM treatment differently affects the lipid profile of hPheo1 and *SDHB*_KD cells (Figure 6 and Figure 7). Understanding the significance of *SDHx*-dependent changes, including lipid metabolism, in PPGL is important and it has consequences broader than solely *SDHx* deficiency. For example, functional succinate dehydrogenase deficiency has been reported to be a common adverse feature of clear cell renal cell carcinoma (ccRCC), the most common type of kidney cancer with poor prognosis [80]. In support of this work, there is additional evidence to suggest that lipid metabolism could be perturbed in PPGL, specifically in instances of tumors carrying SDHx mutations. It has been shown that individuals with *SDHx* deficiency show changes in serum indicating a shift in their FA metabolism, specifically an increase in the elongation of saturated FAs and a higher level of desaturation of FAs in patients with *SDHC/D* variants. The authors also observed a rise in the C20–C24 FAs in patients who have PPGL in combination with *SDHx* genetic deficiency [36]. Furthermore, in a mouse model of *SDHB* loss (pancreatic ß cells deficient in *SDHB* (*SDHB*^ßKO^)), a combination of transcriptomic and metabolomic analysis identified the fatty acid, lipid, and cholesterol metabolism pathways, including SREBP-regulated cholesterol and fatty acid biosynthesis as the most significantly altered in *SDHB*^βKO^ islets [37]. Another notable intersection is between the SDHx-dependent pathway and polyamine metabolism, and it lies in the regulation of T-cell differentiation. The SDHx complex is critical to T-cell regulation and its inhibition leads to a drastic impairment of T-cell proliferation and cytokine secretion. These changes represent an integral part of T-cell functionality and induce a proinflammatory gene signature in T cells, promoting T-helper (T_H_) lineage differentiation [38,39]. Similarly, recent research shows that the polyamine pathway is key to guiding CD4+ helper T_H_ differentiation and function [40,41,42].

Our analyses also demonstrated an increase in the ceramide levels in *SDHB*_KD cells. Several publications have identified mitochondrial ceramide as responsible for apoptosis, hence upregulation of ceramides and cell death can be accountable for DENSPM anti-tumor activity (reviewed in [81]). Ceramides are known to play key roles in cellular signaling during stress response, helping to regulate cellular proliferation and cell death (reviewed in [82,83,84,85,86]). Ceramides can also be generated during mitochondrial apoptosis cascades under stress conditions in some cell lines [87,88]. The exact mechanisms underlying SDHB-related alterations in metabolism are not yet fully understood, and further research is needed to clarify the relationship between the polyamine pathway, lipid metabolism, and PPGL.

## 4. Materials and Methods

### 4.1. RNA Isolation

A total of 1 × 10^5^ hPheo1 and *SDHB*_KD cells were seeded in 60 mm plates and cultured in triplicate until a reaching density of 5 × 10^5^. The cells were either left untreated or treated with 10µM DENSPM. After 3 days, the cells were harvested and total RNA isolated. RNA extraction was performed using TRI^®^ reagent (MilliporeSigma, St. Louis, MO, USA) s per the manufacturer instructions, followed by DNase I treatment (Roche Diagnostics Corporation, Indianapolis, IN, USA) according to the supplier guidelines. The DNase I-treated RNA was further purified using Qiagen Mini columns (Qiagen, Germantown, MD, USA). RNA concentration was initially measured with a NanoDrop Lite spectrophotometer and further with a Qubit^®^ 2.0 Fluorometer (ThermoFisher/Invitrogen, Grand Island, NY, USA) to assure quality control. RNA quality was assessed using the Agilent TapeStation 4200 (Agilent Technologies, Inc., Santa Clara, CA, USA). Only total RNA samples with 28S/18S > 1 and RNA integrity number (RIN) ≥ 7 were used for RNA-seq library preparation. The RIN values for all samples ranged between 7.5 and 9.4.

### 4.2. mRNA RNAseq Library Construction

The RNA libraries were prepared at the Interdisciplinary Center for Biotechnology Research (ICBR) Gene Expression Core, University of Florida, with sequencing conducted at the NextGen core. RNA-seq library preparation utilized 2 μL of 1:200 diluted RNA spike-in from the External RNA Controls Consortium (ERCC; 0.5× recommended amount per the ERCC user guide: Cat# 4456740) and 250 ng of total RNA. mRNA was isolated using NEBNext Poly(A) mRNA Magnetic Isolation module (New England Biolabs, Ipswich, MA, USA catalog # E7490) and RNA library construction followed with NEBNext Ultra II Directional RNA Library Prep Kit (New England Biolabs, catalog # E7530) according to the manufacturer’s protocol. The RNA fragmenting time was adjusted based on the RIN of the total RNA. Specifically, 1000 ng of total RNA along with 2 μL of 1:200 diluted ERCC were incubated with 15 μL of NEBNext Magnetic Oligo d(T)25 and fragmented in an NEBNext First Strand Synthesis Buffer by heating at 94 °C for the appropriate time. First strand cDNA synthesis was carried out with reverse transcriptase and random primers, followed by the second strand synthesis with the provided master mix. The resulting double-stranded DNA was end-repaired, dA-tailed, and ligated with NEBNext adaptors. The libraries were enriched through 13 cycles of amplification and purified using Meg-Bind RxnPure Plus beads (Omega Biotek, Norcross, GA, USA, catalog # M1386). For quality control and pooling, the barcoded libraries were sized using a bioanalyzer and quantitated with QUBIT and qPCR (Kapa Biosystems, Wilmington, MA, USA, catalog number: KK4824). Fifteen barcoded libraries were further sized using the TapeStation 4200 and quantified with the Qubit^®^ 2.0 Fluorometer. Barcoded libraries were then pooled equimolarly and sequenced simultaneously with NavaSeq 6000 S4 2 × 150 cycles run. RNA-seq library preparation was conducted at UF ICBR Gene Expression Core (https://biotech.ufl.edu/gene-expression-genotyping/, RRID:SCR_019145 15 January 2023).

### 4.3. Illumina NovaSeq6000 Sequencing and Analysis

The normalized libraries were processed using the “Free Adapter Blocking Reagent” protocol (FAB, Cat# 20024145) to reduce adaptor–dimer formation and minimize index hopping rates. The library pool was diluted to 0.8 nM and sequenced on one S4 flow cell lane (2 × 150 cycles) of the Illumina NovaSeq6000. The instrument used the NovaSeq Control Software v1.6. Cluster and SBS consumables were v1.5. The final library loading was at 120 pM with a 1% PhiX spike-in control. A single lane produced 2.5–3 billion paired-end reads (~950 Gb) with an average Q30% >= 92.5% and Cluster PF = 85.4%. The Illumina NovaSeq 6000 was used to sequence the libraries for 2 × 150 cycles. Sequencing was carried out at ICBR NextGen Sequencing (https://biotech.ufl.edu/next-gen-dna/, RRID:SCR_019152 accessed on 24 June 2023). A total of 12 individual libraries were pooled at equimolar concentration of 20 nM and two lanes of HiSeq 000 were run. FastQ files were generated using the BCL2fastQ function in the Illumina BaseSpace portal.

The sequencing reads underwent quality control filtering and were mapped to the reference Human Genome 38 (GRCh 38.p13). Differentially expressed genes were identified using Deseq2 package with an adjusted *p* value < 0.05 and cutoff parameters |FC| >= 2 and FDR < 0.05. These genes were visualized on a volcano plot, with those Qval < 4.78 × 10^−95^ and log2 (fold change) > 5 assigned and labeled. To perform GSEA analysis a gene list (h.all.v2023.2.Hs.symbols.gmt file) was obtained from https://www.gsea-msigdb.org/gsea/msigdb/collections.jsp (accessed on 11 January 2024) and differentially expressed genes were filtered for FC > 1.5 to reduces the noise. Further, analysis of differentially expressed genes was conducted using The Database for Annotation, Visualization and Integrated Discovery (DAVID; https://david.ncifcrf.gov accessed on 11 January 2024) for KEGG pathway analysis. Additionally, the gene set was examined using Gene Set Enrichment Analysis (GSEA, https://www.gsea-msigdb.org/gsea/index.jsp accessed on 16 January 2024), and the Molecular Signatures Database (MSigDB). We noted that the fatty acid metabolism gene set is among the three enriched sets with a *p*-value below < 0.01 (out of total 40), determined by GSEA. Differential expression of genes involved in de novo FA synthesis and FA import pathways was also analyzed by qRT-PCR as detailed below.

### 4.4. Quantitative RT-PCR (qPCR)

For reverse transcription, equal amounts of total RNA were converted into cDNA using the M-MLV (Moloney Murine Leukemia Virus) Reverse Transcriptase kit (Invitrogen, Thermo), following the manufacturer’s instructions. Quantitative RT-PCR was performed on an LC480 system (Roche Diagnostics Corporation, Indianapolis, USA) with SYBR Green PCR master mix (Applied Biosystems, Foster City, CA, USA). The PCR cycling conditions were as follows: 40 cycles at 95 °C for 15 s and 60 °C for 1 min, preceded by an initial step of 2 min at 50 °C and 10 min at 95 °C. Primer sequences are listed in Table 1. Gene expression was normalized using the ACTB (human beta actin) gene RNA which was confirmed to be stable based on RNAseq data.

Each sample was tested in triplicate across at least three independent biological experiments and gene expression changes were calculated using the ∆∆Ct method. Statistical significance of ∆∆Ct values was determined by the Mann–Whitney test, with *p* < 0.05 considered significant. Results were presented as fold-change differences relative to wild-type controls using GraphPad Prism^®^ software, version 6.02 (San Diego, CA, USA). A fold change of 1 indicates no change in gene expression.

### 4.5. Western Blotting Analysis

Whole cell lysates were generated using a sodium deoxycholate lysis buffer, while nuclear protein extracts were obtained using a dual-buffer method, with the first buffer containing a detergent followed by the second buffer containing glycerol. Samples were derived from well-washed cell pellets, either from control or treated cells, which were flash-frozen and stored at −80 °C. Protein concentrations were measured with a NanoDrop Lite spectrophotometer (ThermoFisher Scientific Inc., Waltham, MA, USA). A 100 µL aliquot from each sample was boiled with 4X LDS sample buffer (Invitrogen) for 5 min. A total of 30 µg of protein per sample was loaded and separated on a 12% SDS-PAGE gel along with a BenchMark Protein Ladder (Invitrogen). Proteins were transferred onto a PVDF (BioRad, Hercules, CA, USA) membrane. The membranes were incubated with either sheep anti-hSCD1 antibody (R&D Systems, Minneapolis, MN, USA), rabbit anti-hFADS2 antibody (Proteintech, Rosemont, IL, USA), or mouse anti-hSREBP1 antibody (Santa Cruz Biotechnology, Inc., Dallas, TX, USA) followed by respective anti-sheep, anti-rabbit, or anti-mouse HRP secondary antibodies, respectively (BioRad). The HRP signal was developed using Clarify Western ECL substrate (BioRad) and detected with a Li-Cor scanner and Image Studio Digits version 3.1. To confirm equal sample loading, the membranes were probed with either anti-beta actin (ACTB) or alpha-actinin-1 (ACTN1) antibodies (Novus, Centennial, CO, USA), followed by anti-mouse HRP secondary antibody and ECL development and detection.

### 4.6. Liquid Chromatography—Mass Spectrometry

Supernatants (500 mL) and cell pellets (in 100 mL PBS) were weighed using a gravimetric method and 40 mL of a 1:10 dilution of EquiSPLASH Lipidomix (stable isotope-labelled internal standards, Avanti Polar Lipids; Alabaster, AL, USA) in methanol was added to each sample. Lipid extraction was performed using the Folch method [89]. Extraction blanks, both with and without internal standards, along with 3 replicates of NIST SRM 1950-Metabolites in Frozen Human Plasma were included to monitor instrument performance and check for background contamination. To prevent bias, all lipid extracts, blanks, and quality control samples were randomized throughout the analysis sequence.

The untargeted ultra high-performance liquid chromatography–tandem mass spectrometric (UHPLC-MS/MS) lipidomic analysis was conducted using a Thermo Scientific Vanquish Horizon UHPLC system with a Thermo Scientific Accucore C30 column (2.1 mm × 150 mm × 2.6 μm) coupled to a Thermo Scientific Q Exactive Orbitrap series mass spectrometer (Waltham, MA, USA). The gradient ramp was applied using mobile phase A (60:40 acetonitrile:water (*v*/*v*)) and mobile phase B (90:10 isopropanol:acetonitrile (*v*/*v*)), both with 5 mmol/L ammonium formate and 0.1% formic acid. The gradient started at 40% B, increased linearly to 55% B at 7 min, held at 65% B from 8 min to 12 min, then increased to 95% B at 20 min, reaching 100% B at 22 min and held until 27 min. At 27.1 min, the gradient was reduced back to 40% and held until the end of the method. The flow rate was maintained at 0.400 mL/min and the column compartment was kept at 45 °C throughout the run. All samples were analyzed in full-scan mode under positive and negative electrospray ionization conditions with an injection volume of 10 μL. The spray voltage was set at +/−3.5 kV, with capillary temperatures of 320 °C and 275 °C for both positive and negative modes, respectively. The scan range was 200–1200 *m*/*z*, and the resolution was set at 35,000. Tandem mass spectra were acquired by pooling samples for each matrix type (cells and supernatant), using a top-10 data-dependent acquisition method with a resolution of 17,500, isolation window of 1.0 *m*/*z*, dynamic exclusion of 6 s, and stepped-normalized collision energy at 20, 25, and 30 eVs. IE-omics [90] was used to generate iterative exclusion lists in three consecutive rounds for both positive and negative ionization modes.

Lipid identification and peak integration were carried out using LipidMatch Flow (v3.5) [91]. The precursor ion *m*/*z* tolerance was set at ±0.5 mDa, product ion *m*/*z* tolerance was ±5 ppm, and retention time tolerance was ±0.07 min. Peak areas were normalized based on labeled internal standards corresponding to the lipid class of the identified features. For compounds lacking an internal standard of the same subclass, the standard with the closest chemical structure was used. If this was not feasible, the internal standard with the closest retention time in the same polarity was applied. The quantified lipids were normalized by sample mass, and concentrations expressed in mg/g. (All lipid concentrations are listed in the Supplemental Excel Table).

## 5. Conclusions

In this study we explored the mechanism behind the action of the polyamine analogue drug DENSPM in hPheo1 cells derived from PCC. RNAseq analyses of both the hPheo1 cell line and its SDHB_KD derivative identified biochemical pathways linked to DENSPM treatment that broadly disrupt various aspects of lipid metabolism, including fatty acid (FA) synthesis, desaturation, and import/uptake. Additionally, untargeted lipidomic analysis using liquid chromatography–mass spectrometry (LC/MS) revealed a specific group of lipids, ether lipids, which are dramatically reduced following DENSPM treatment. Ether lipids have been described to act in cancer cells as crucial gatekeepers of metastatic progression while at the same time serving as a conduit for ferroptotic death that can be induced by therapeutic intervention. This work suggests a novel role for polyamines in regulating lipid metabolism in neuroendocrine cells and tumors.

## Figures and Tables

**Table 1:** Real Time PCR Primers.

FADS2_F	TGACCGCAAGGTTTACAACAT	FADS2_R	AGGCATCCGTTGCATCTTCTC	[17]
SCD1_F	CCGGGAGAATATCCTGGTTT	SCD1_R	GCGGTACTCAACTGGCAGAGT	[18]
SREBF1_F	ACAGTGACTTCCCTGGCCTAT	SREBF1_R	GCATGGACGGGTACATCTTCAA	[19]
SREBF2_F	CCTGGGAGACATCGACGAGAT	SREBF2_R	TGAATGACCGTTGCACTGAAG	[20]
ACTB_F	CACCATTGGCAATGAGCGGTTC	ACTB_R	AGGTCTTTGCGGATGTCCACGT	[21]

## Data Availability

Data will be available in a publicly accessible repository upon the manuscript’ acceptance.

## Supplementary Materials

The following supporting information can be downloaded at: https://www.mdpi.com/article/10.3390/ijms251810029/s1. References [92,93,94,95,96] are cited in the supplementary materials.

## References

[1] Tevosian S.G., Ghayee H.K. (2019). Pheochromocytomas and Paragangliomas. Endocrinol. Metab. Clin. N. Am..

[2] Taïeb D., Wanna G.B., Ahmad M., Lussey-Lepoutre C., Perrier N.D., Nölting S., Amar L., Timmers H.J.L.M., Schwam Z.G., Estrera A.L. (2023). Clinical consensus guideline on the management of phaeochromocytoma and paraganglioma in patients harbouring germline SDHD pathogenic variants. Lancet Diabetes Endocrinol..

[3] Mete O., Asa S.L., Gill A.J., Kimura N., de Krijger R.R., Tischler A. (2022). Overview of the 2022 WHO Classification of Paragangliomas and Pheochromocytomas. Endocr. Pathol..

[4] Fishbein L., Leshchiner I., Walter V., Danilova L., Robertson A.G., Johnson A.R., Lichtenberg T.M., Murray B.A., Ghayee H.K., Else T. (2017). Comprehensive Molecular Characterization of Pheochromocytoma and Paraganglioma. Cancer Cell.

[5] Turin C.G., Crenshaw M.M., Fishbein L. (2022). Pheochromocytoma and paraganglioma: Germline genetics and hereditary syndromes. Endocr. Oncol..

[6] Ghayee H.K., Bhagwandin V.J., Stastny V., Click A., Ding L.-H., Mizrachi D., Zou Y.S., Chari R., Lam W.L., Bachoo R.M. (2013). Progenitor Cell Line (hPheo1) Derived from a Human Pheochromocytoma Tumor. PLoS ONE.

[7] Rai S.K., Bril F., Hatch H.M., Xu Y., Shelton L., Kalavalapalli S., Click A., Lee D., Beecher C., Kirby A. (2020). Targeting pheochromocytoma/paraganglioma with polyamine inhibitors. Metabolism.

[8] Kramer D.L., Chang B.D., Chen Y., Diegelman P., Alm K., Black A.R., Roninson I.B., Porter C.W. (2001). Polyamine depletion in human melanoma cells leads to G1 arrest associated with induction of p21WAF1/CIP1/SDI1, changes in the expression of p21-regulated genes, and a senescence-like phenotype. Cancer Res..

[9] Casero R.A., Stewart T.M., Pegg A.E. (2018). Polyamine metabolism and cancer: Treatments, challenges and opportunities. Nat. Rev. Cancer.

[10] Li J., Meng Y., Wu X., Sun Y. (2020). Polyamines and related signaling pathways in cancer. Cancer Cell Int..

[11] Khan A., Gamble L.D., Upton D.H., Ung C., Yu D.M.T., Ehteda A., Pandher R., Mayoh C., Hébert S., Jabado N. (2021). Dual targeting of polyamine synthesis and uptake in diffuse intrinsic pontine gliomas. Nat. Commun..

[12] Gamble L.D., Purgato S., Murray J., Xiao L., Yu D.M.T., Hanssen K.M., Giorgi F.M., Carter D.R., Gifford A.J., Valli E. (2019). Inhibition of polyamine synthesis and uptake reduces tumor progression and prolongs survival in mouse models of neuroblastoma. Sci. Transl. Med..

[13] Bi G., Liang J., Bian Y., Shan G., Huang Y., Lu T., Zhang H., Jin X., Chen Z., Zhao M. (2024). Polyamine-mediated ferroptosis amplification acts as a targetable vulnerability in cancer. Nat. Commun..

[14] Lee M.-S., Dennis C., Naqvi I., Dailey L., Lorzadeh A., Ye G., Zaytouni T., Adler A., Hitchcock D.S., Lin L. (2023). Ornithine aminotransferase supports polyamine synthesis in pancreatic cancer. Nature.

[15] Snaebjornsson M.T., Janaki-Raman S., Schulze A. (2020). Greasing the Wheels of the Cancer Machine: The Role of Lipid Metabolism in Cancer. Cell Metab..

[16] Zhang C., Zhu N., Li H., Gong Y., Gu J., Shi Y., Liao D., Wang W., Dai A., Qin L. (2022). New dawn for cancer cell death: Emerging role of lipid metabolism. Mol. Metab..

[17] Korbecki J., Gutowska I., Wiercioch M., Łukomska A., Tarnowski M., Drozd A., Barczak K., Chlubek D., Baranowska-Bosiacka I. (2019). Sodium Orthovanadate Changes Fatty Acid Composition and Increased Expression of Stearoyl-Coenzyme A Desaturase in THP-1 Macrophages. Biol. Trace Element Res..

[18] Zhang Y., Wang H., Zhang J., Lv J., Huang Y. (2013). Positive feedback loop and synergistic effects between hypoxia-inducible factor-2alpha and stearoyl-CoA desaturase-1 promote tumorigenesis in clear cell renal cell carcinoma. Cancer Sci..

[19] Yang H., Zhang X., Liu F., Fan J., Wang B., Dong C. (2018). SREBP1-driven lipid desaturation supports clear cell renal cell carcinoma growth through regulation of NF-kappaB signaling. Biochem. Biophys. Res. Commun..

[20] Zhai Q., Luo M., Zhang Y., Zhang W., Wu C., Lv S., Wei Q. (2022). Histone methyltransferase KMT2D mediated lipid metabolism via peroxisome proliferator-activated receptor gamma in prostate cancer. Transl. Cancer Res..

[21] Ye J., Liang R., Bai T., Lin Y., Mai R., Wei M., Ye X., Li L., Wu F. (2018). RBM38 plays a tumor-suppressor role via stabilizing the p53-mdm2 loop function in hepatocellular carcinoma. J. Exp. Clin. Cancer Res..

[22] Subramanian C., Cohen M.S. (2021). Identification of novel lipid metabolic biomarkers associated with poor adrenocortical carcinoma prognosis using integrated bioinformatics. Surgery.

[23] Duranova H., Fialkova V., Valkova V., Bilcikova J., Olexikova L., Lukac N., Massanyi P., Knazicka Z. (2022). Human adrenocortical carcinoma cell line (NCI-H295R): An in vitro screening model for the assessment of endocrine disruptors’ actions on steroidogenesis with an emphasis on cell ultrastructural features. Acta Histochem..

[24] Warde K.M., Lim Y.J., Martinez E.R., Beuschlein F., O’shea P., Hantel C., Dennedy M.C. (2022). Mitotane Targets Lipid Droplets to Induce Lipolysis in Adrenocortical Carcinoma. Endocrinology.

[25] LaPensee C.R., Hammer G.D. (2023). Targeting of a New Node in Lipid Metabolism as a Potential Treatment Strategy for ACC. Endocrinology.

[26] Broadfield L.A., Pane A.A., Talebi A., Swinnen J.V., Fendt S.-M. (2021). Lipid metabolism in cancer: New perspectives and emerging mechanisms. Dev. Cell.

[27] Martin-Perez M., Urdiroz-Urricelqui U., Bigas C., Benitah S.A. (2022). The role of lipids in cancer progression and metastasis. Cell Metab..

[28] Altea-Manzano P., Doglioni G., Liu Y., Cuadros A.M., Nolan E., Fernández-García J., Wu Q., Planque M., Laue K.J., Cidre-Aranaz F. (2023). A palmitate-rich metastatic niche enables metastasis growth via p65 acetylation resulting in pro-metastatic NF-kappaB signaling. Nat. Cancer.

[29] Kobayashi N., Barnard R.J., Said J., Hong-Gonzalez J., Corman D.M., Ku M., Doan N.B., Gui D., Elashoff D., Cohen P. (2008). Effect of Low-Fat Diet on Development of Prostate Cancer and Akt Phosphorylation in the Hi-Myc Transgenic Mouse Model. Cancer Res..

[30] Labbé D.P., Zadra G., Yang M., Reyes J.M., Lin C.Y., Cacciatore S., Ebot E.M., Creech A.L., Giunchi F., Fiorentino M. (2019). High-fat diet fuels prostate cancer progression by rewiring the metabolome and amplifying the MYC program. Nat. Commun..

[31] Janda C.Y., Garcia K.C. (2015). Wnt acylation and its functional implication in Wnt signalling regulation. Biochem. Soc. Trans..

[32] Henis Y.I., Hancock J.F., Prior I.A. (2009). Ras acylation, compartmentalization and signaling nanoclusters (Review). Mol. Membr. Biol..

[33] Jarc E., Petan T. (2019). Lipid Droplets and the Management of Cellular Stress. Yale J. Biol. Med..

[34] Batlle E., Clevers H. (2017). Cancer stem cells revisited. Nat. Med..

[35] Faubert B., Solmonson A., DeBerardinis R.J. (2020). Metabolic reprogramming and cancer progression. Science.

[36] Vamecq J., Masso V., Bancel L.-P., Jannin A., Dessein A.-F., Cardot-Bauters C., Pigny P. (2022). Serum fatty acid profiling in patients with SDHx mutations: New advances on cellular metabolism in SDH deficiency. Biochimie.

[37] Lee S., Xu H., Van Vleck A., Mawla A.M., Li A.M., Ye J., Huising M.O., Annes J.P. (2022). Beta-Cell Succinate Dehydrogenase Deficiency Triggers Metabolic Dysfunction and Insulinopenic Diabetes. Diabetes.

[38] Chen X., Sunkel B., Wang M., Kang S., Wang T., Gnanaprakasam J.N.R., Liu L., Cassel T.A., Scott D.A., Muñoz-Cabello A.M. (2022). Succinate dehydrogenase/complex II is critical for metabolic and epigenetic regulation of T cell proliferation and inflammation. Sci. Immunol..

[39] Nastasi C., Willerlev-Olsen A., Dalhoff K., Ford S.L., Gadsbøll A.-S., Buus T.B., Gluud M., Danielsen M., Litman T., Bonefeld C.M. (2021). Inhibition of succinate dehydrogenase activity impairs human T cell activation and function. Sci. Rep..

[40] Puleston D.J., Baixauli F., Sanin D.E., Edwards-Hicks J., Villa M., Kabat A.M., Kamiński M.M., Stanckzak M., Weiss H.J., Grzes K.M. (2021). Polyamine metabolism is a central determinant of helper T cell lineage fidelity. Cell.

[41] Wang Z. (2021). Polyamines instruct T-cell differentiation. Nat. Cell Biol..

[42] Wagner A., Wang C., Fessler J., DeTomaso D., Avila-Pacheco J., Kaminski J., Zaghouani S., Christian E., Thakore P., Schellhaass B. (2021). Metabolic modeling of single Th17 cells reveals regulators of autoimmunity. Cell.

[43] Menendez J.A., Lupu R. (2007). Fatty acid synthase and the lipogenic phenotype in cancer pathogenesis. Nat. Rev. Cancer.

[44] Medes G., Thomas A., Weinhouse S. (1953). Metabolism of neoplastic tissue. IV. A study of lipid synthesis in neoplastic tissue slices in vitro. Cancer Res..

[45] Ookhtens M., Kannan R., Lyon I., Baker N. (1984). Liver and adipose tissue contributions to newly formed fatty acids in an ascites tumor. Am. J. Physiol. Integr. Comp. Physiol..

[46] Swinnen J.V., Brusselmans K., Verhoeven G. (2006). Increased lipogenesis in cancer cells: New players, novel targets. Curr. Opin. Clin. Nutr. Metab. Care.

[47] Röhrig F., Schulze A. (2016). The multifaceted roles of fatty acid synthesis in cancer. Nat. Rev. Cancer.

[48] Cheng C., Geng F., Cheng X., Guo D. (2018). Lipid metabolism reprogramming and its potential targets in cancer. Cancer Commun..

[49] Peck B., Schulze A. (2016). Lipid desaturation—The next step in targeting lipogenesis in cancer?. FEBS J..

[50] Vriens K., Christen S., Parik S., Broekaert D., Yoshinaga K., Talebi A., Dehairs J., Escalona-Noguero C., Schmieder R., Cornfield T. (2019). Evidence for an alternative fatty acid desaturation pathway increasing cancer plasticity. Nature.

[51] Dierge E., Feron O. (2019). Dealing with saturated and unsaturated fatty acid metabolism for anticancer therapy. Curr. Opin. Clin. Nutr. Metab. Care.

[52] Corbet C., Feron O. (2017). Emerging roles of lipid metabolism in cancer progression. Curr. Opin. Clin. Nutr. Metab. Care.

[53] Fhu C.W., Ali A. (2020). Fatty Acid Synthase: An Emerging Target in Cancer. Molecules.

[54] Menendez J.A., Lupu R. (2017). Fatty acid synthase (FASN) as a therapeutic target in breast cancer. Expert. Opin. Ther. Targets.

[55] Dierge E., Larondelle Y., Feron O. (2020). Cancer diets for cancer patients: Lessons from mouse studies and new insights from the study of fatty acid metabolism in tumors. Biochimie.

[56] Oatman N., Dasgupta N., Arora P., Choi K., Gawali M.V., Gupta N., Parameswaran S., Salomone J., Reisz J.A., Lawler S. (2021). Mechanisms of stearoyl CoA desaturase inhibitor sensitivity and acquired resistance in cancer. Sci. Adv..

[57] Guo Z., Bergeron K.-F., Lingrand M., Mounier C. (2023). Unveiling the MUFA–Cancer Connection: Insights from Endogenous and Exogenous Perspectives. Int. J. Mol. Sci..

[58] Morais C.M., Cardoso A.M., Araújo A.R.D., Reis A., Domingues P., Domingues M.R.M., de Lima M.C.P., Jurado A.S. (2022). Stearoyl CoA Desaturase-1 Silencing in Glioblastoma Cells: Phospholipid Remodeling and Cytotoxicity Enhanced upon Autophagy Inhibition. Int. J. Mol. Sci..

[59] Parik S., Fernández-García J., Lodi F., De Vlaminck K., Derweduwe M., De Vleeschouwer S., Sciot R., Geens W., Weng L., Bosisio F.M. (2022). GBM tumors are heterogeneous in their fatty acid metabolism and modulating fatty acid metabolism sensitizes cancer cells derived from recurring GBM tumors to temozolomide. Front. Oncol..

[60] Huang J., Fan X.-X., He J., Pan H., Li R.Z., Huang L., Jiang Z., Yao X.-J., Liu L., Leung E.L. (2016). SCD1 is associated with tumor promotion, late stage and poor survival in lung adenocarcinoma. Oncotarget.

[61] She K., Fang S., Du W., Fan X., He J., Pan H., Huang L., He P., Huang J. (2019). SCD1 is required for EGFR-targeting cancer therapy of lung cancer via re-activation of EGFR/PI3K/AKT signals. Cancer Cell Int..

[62] Jafari N., Drury J., Morris A.J., Onono F.O., Stevens P.D., Gao T., Liu J., Wang C., Lee E.Y., Weiss H.L. (2019). *De. Novo* Fatty Acid Synthesis-Driven Sphingolipid Metabolism Promotes Metastatic Potential of Colorectal Cancer. Mol. Cancer Res..

[63] Tesfay L., Paul B.T., Konstorum A., Deng Z., Cox A.O., Lee J., Furdui C.M., Hegde P., Torti F.M., Torti S.V. (2019). Stearoyl-CoA Desaturase 1 Protects Ovarian Cancer Cells from Ferroptotic Cell Death. Cancer Res..

[64] Triki M., Rinaldi G., Planque M., Broekaert D., Winkelkotte A.M., Maier C.R., Raman S.J., Vandekeere A., Van Elsen J., Orth M.F. (2020). mTOR Signaling and SREBP Activity Increase FADS2 Expression and Can Activate Sapienate Biosynthesis. Cell Rep..

[65] Munir R., Lisec J., Swinnen J.V., Zaidi N. (2019). Lipid metabolism in cancer cells under metabolic stress. Br. J. Cancer.

[66] Kamphorst J.J., Cross J.R., Fan J., de Stanchina E., Mathew R., White E.P., Thompson C.B., Rabinowitz J.D. (2013). Hypoxic and Ras-transformed cells support growth by scavenging unsaturated fatty acids from lysophospholipids. Proc. Natl. Acad. Sci. USA.

[67] Bensaad K., Favaro E., Lewis C.A., Peck B., Lord S., Collins J.M., Pinnick K.E., Wigfield S., Buffa F.M., Li J.-L. (2014). Fatty Acid Uptake and Lipid Storage Induced by HIF-1α Contribute to Cell Growth and Survival after Hypoxia-Reoxygenation. Cell Rep..

[68] Lewis C.A., Brault C., Peck B., Bensaad K., Griffiths B., Mitter R., Chakravarty P., East P., Dankworth B., Alibhai D. (2015). SREBP maintains lipid biosynthesis and viability of cancer cells under lipid- and oxygen-deprived conditions and defines a gene signature associated with poor survival in glioblastoma multiforme. Oncogene.

[69] Gharpure K.M., Pradeep S., Sans M., Rupaimoole R., Ivan C., Wu S.Y., Bayraktar E., Nagaraja A.S., Mangala L.S., Zhang X. (2018). FABP4 as a key determinant of metastatic potential of ovarian cancer. Nat. Commun..

[70] Gallagher E.J., Zelenko Z., Neel B.A., Antoniou I.M., Rajan L., Kase N., LeRoith D. (2017). Elevated tumor LDLR expression accelerates LDL cholesterol-mediated breast cancer growth in mouse models of hyperlipidemia. Oncogene.

[71] Floeth M., Elges S., Gerss J., Schwöppe C., Kessler T., Herold T., Wardelmann E., Berdel W.E., Lenz G., Mikesch J. (2020). Low-density lipoprotein receptor (LDLR) is an independent adverse prognostic factor in acute myeloid leukaemia. Br. J. Haematol..

[72] Gonias S.L., Karimi-Mostowfi N., Murray S.S., Mantuano E., Gilder A.S. (2017). Expression of LDL receptor-related proteins (LRPs) in common solid malignancies correlates with patient survival. PLoS ONE.

[73] Guillaumond F., Bidaut G., Ouaissi M., Servais S., Gouirand V., Olivares O., Lac S., Borge L., Roques J., Gayet O. (2015). Cholesterol uptake disruption, in association with chemotherapy, is a promising combined metabolic therapy for pancreatic adenocarcinoma. Proc. Natl. Acad. Sci. USA.

[74] Bovenga F., Sabbà C., Moschetta A. (2015). Uncoupling Nuclear Receptor LXR and Cholesterol Metabolism in Cancer. Cell Metab..

[75] Pascual G., Avgustinova A., Mejetta S., Martín M., Castellanos A., Attolini C.S.-O., Berenguer A., Prats N., Toll A., Hueto J.A. (2017). Targeting metastasis-initiating cells through the fatty acid receptor CD36. Nature.

[76] Ruiz C.F., Montal E.D., Bott A.J., Haley J.D. (2020). SREBP1 regulates mitochondrial metabolism in oncogenic *KRAS* expressing NSCLC. FASEB J..

[77] Snyder F., Wood R. (1969). Alkyl and alk-1-enyl ethers of glycerol in lipids from normal and neoplastic human tissues. Cancer Res..

[78] Benjamin D.I., Cozzo A., Ji X., Roberts L.S., Louie S.M., Mulvihill M.M., Luo K., Nomura D.K. (2013). Ether lipid generating enzyme AGPS alters the balance of structural and signaling lipids to fuel cancer pathogenicity. Proc. Natl. Acad. Sci. USA.

[79] Henry W.S., Müller S., Yang J.S., Innes-Gold S., Das S., Reinhardt F., Sigmund K., Phadnis V.V., Wan Z., Eaton E. Ether lipids influence cancer cell fate by modulating iron uptake. bioRxiv.

[80] Aggarwal R.K., Luchtel R.A., Machha V., Tischer A., Zou Y., Pradhan K., Ashai N., Ramachandra N., Albanese J.M., Yang J.-I. (2021). Functional succinate dehydrogenase deficiency is a common adverse feature of clear cell renal cancer. Proc. Natl. Acad. Sci. USA.

[81] Wajapeyee N., Beamon T.C., Gupta R. (2024). Roles and therapeutic targeting of ceramide metabolism in cancer. Mol. Metab..

[82] Canals D., Hannun Y.A. (2024). Biological function, topology, and quantification of plasma membrane Ceramide. Adv. Biol. Regul..

[83] Hannun Y.A., Obeid L.M. (2011). Many ceramides. J. Biol. Chem..

[84] Hannun Y.A., Luberto C. (2000). Ceramide in the eukaryotic stress response. Trends Cell Biol..

[85] Ruvolo P.P. (2001). Ceramide regulates cellular homeostasis via diverse stress signaling pathways. Leukemia.

[86] Pettus B.J., Chalfant C.E., Hannun Y.A. (2002). Ceramide in apoptosis: An overview and current perspectives. Biochim. Biophys. Acta (BBA)-Mol. Cell Biol. Lipids.

[87] Gudz T.I., Tserng K.-Y., Hoppel C.L. (1997). Direct Inhibition of Mitochondrial Respiratory Chain Complex III by Cell-permeable Ceramide. J. Biol. Chem..

[88] Claus R., Dorer M., Bunck A., Deigner H. (2009). Inhibition of Sphingomyelin Hydrolysis: Targeting the Lipid Mediator Ceramide as a Key Regulator of Cellular Fate. Curr. Med. Chem..

[89] Folch J., Lees M., Stanley G.H.S. (1957). A simple method for the isolation and purification of total lipides from animal tissues. J. Biol. Chem..

[90] Koelmel J.P., Kroeger N.M., Gill E.L., Ulmer C.Z., Bowden J.A., Patterson R.E., Yost R.A., Garrett T.J. (2017). Expanding Lipidome Coverage Using LC-MS/MS Data-Dependent Acquisition with Automated Exclusion List Generation. J. Am. Soc. Mass. Spectrom..

[91] Koelmel J.P., Kroeger N.M., Ulmer C.Z., Bowden J.A., Patterson R.E., Cochran J.A., Beecher C.W.W., Garrett T.J., Yost R.A. (2017). LipidMatch: An automated workflow for rule-based lipid identification using untargeted high-resolution tandem mass spectrometry data. BMC Bioinform..

[92] Li J., Dong L., Wei D., Wang X., Zhang S., Li H. (2014). Fatty acid synthase mediates the epithelial-mesenchymal transition of breast cancer cells. Int. J. Biol. Sci..

[93] Veprik A., Denwood G., Liu D., Bany Bakar R., Morfin V., McHugh K., Tebeka N.N., Vetterli L., Yonova-Doing E., Gribble F. (2022). Acetyl-CoA-carboxylase 1 (ACC1) plays a critical role in glucagon secretion. Commun. Biol..

[94] Kolahi K., Louey S., Varlamov O., Thornburg K. (2016). Real-Time Tracking of BODIPY-C12 Long-Chain Fatty Acid in Human Term Placenta Reveals Unique Lipid Dynamics in Cytotrophoblast Cells. PLoS ONE.

[95] Fernández L.P., Merino M., Colmenarejo G., Moreno-Rubio J., Sánchez-Martínez R., Quijada-Freire A., Gomez de Cedron M., Reglero G., Casado E., Sereno M. (2020). Metabolic enzyme ACSL3 is a prognostic biomarker and correlates with anticancer effectiveness of statins in non-small cell lung cancer. Mol. Oncol..

[96] Pérez-Núñez I., Karaky M., Fedetz M., Barrionuevo C., Izquierdo G., Matesanz F., Alcina A. (2019). Splice-site variant in ACSL5: A marker promoting opposing effect on cell viability and protein expression. Eur. J. Hum. Genet..

