# Supplementary material for: Polyamine Pathway Inhibitor DENSPM Suppresses Lipid Metabolism in Pheochromocytoma Cell Line
Source: Int J Mol Sci. 2024 Sep 18;25(18):10029. doi: 10.3390/ijms251810029 (PMC11432427; doi:10.3390/ijms251810029)
Supplement: Supplementary file 1 [file ijms-25-10029-s001.zip › Editable_Supplemental_IJMS_09_17.pptx]

## Slide 1
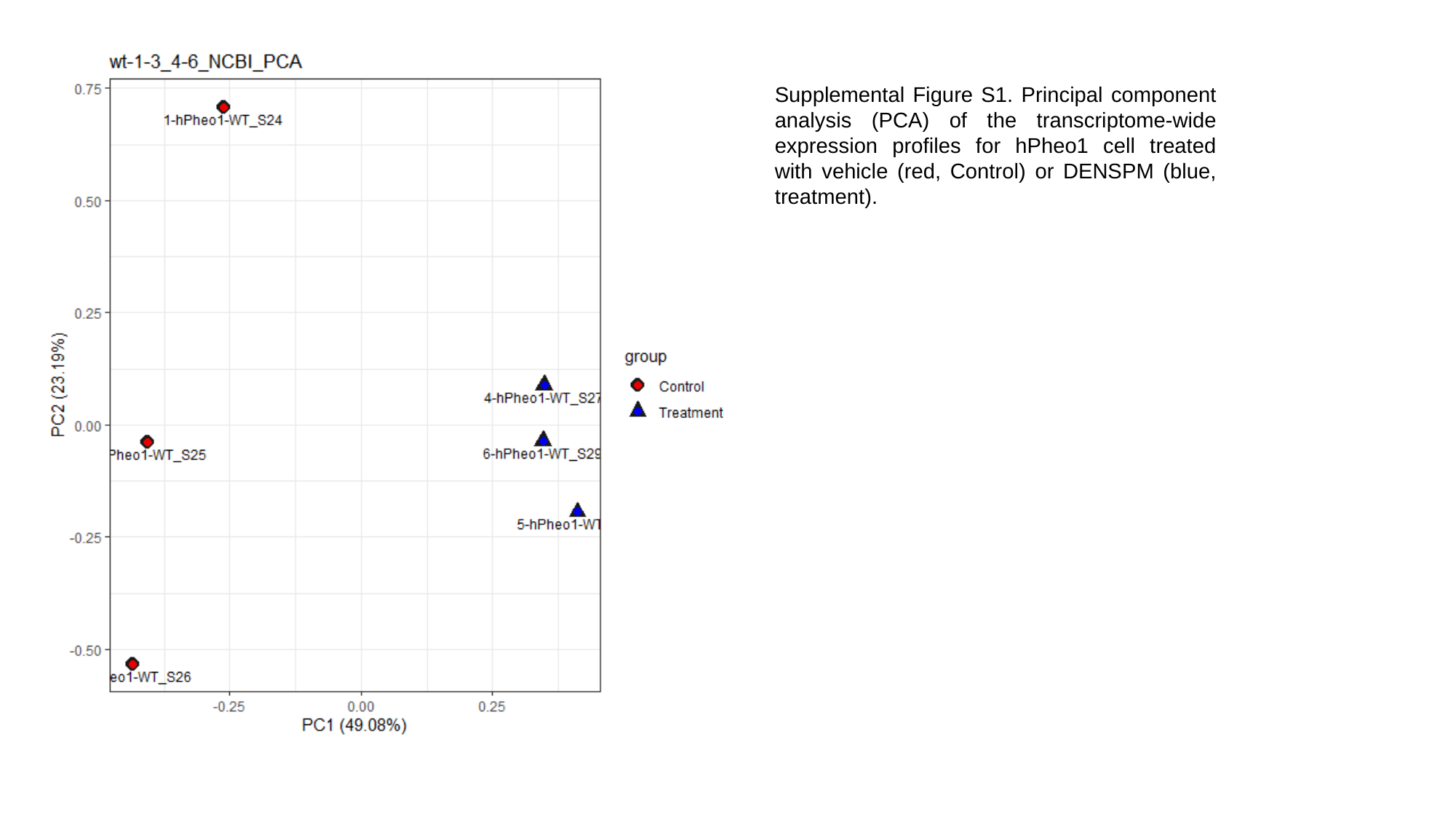

Supplemental Figure S1. Principal component analysis (PCA) of the transcriptome-wide expression profiles for hPheo1 cell treated with vehicle (red, Control) or DENSPM (blue, treatment).

## Slide 2
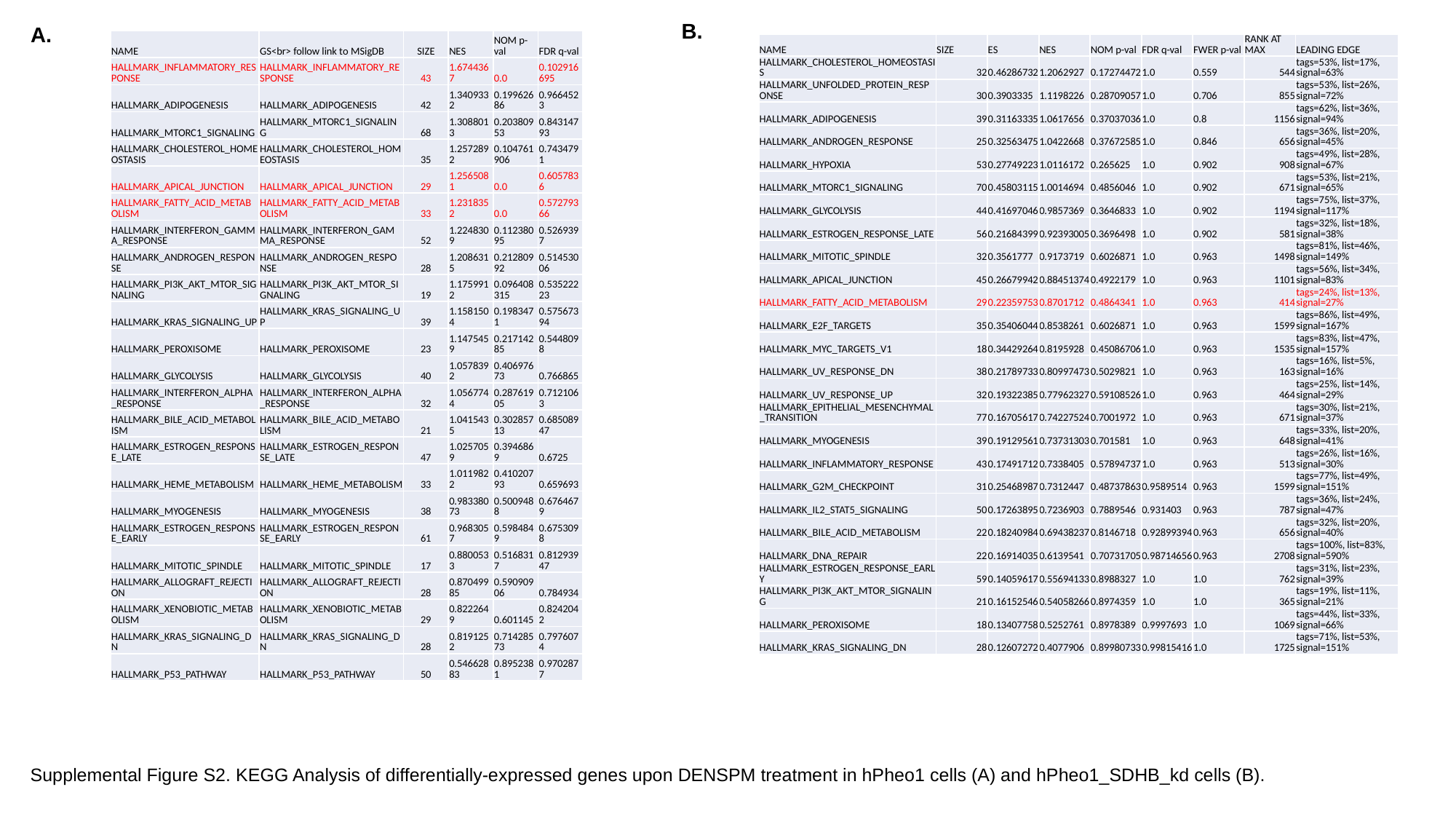

B.
A.
| NAME | GS<br> follow link to MSigDB | SIZE | NES | NOM p-val | FDR q-val |
| --- | --- | --- | --- | --- | --- |
| HALLMARK\_INFLAMMATORY\_RESPONSE | HALLMARK\_INFLAMMATORY\_RESPONSE | 43 | 1.6744367 | 0.0 | 0.102916695 |
| HALLMARK\_ADIPOGENESIS | HALLMARK\_ADIPOGENESIS | 42 | 1.3409332 | 0.19962686 | 0.9664523 |
| HALLMARK\_MTORC1\_SIGNALING | HALLMARK\_MTORC1\_SIGNALING | 68 | 1.3088013 | 0.20380953 | 0.84314793 |
| HALLMARK\_CHOLESTEROL\_HOMEOSTASIS | HALLMARK\_CHOLESTEROL\_HOMEOSTASIS | 35 | 1.2572892 | 0.104761906 | 0.7434791 |
| HALLMARK\_APICAL\_JUNCTION | HALLMARK\_APICAL\_JUNCTION | 29 | 1.2565081 | 0.0 | 0.6057836 |
| HALLMARK\_FATTY\_ACID\_METABOLISM | HALLMARK\_FATTY\_ACID\_METABOLISM | 33 | 1.2318352 | 0.0 | 0.57279366 |
| HALLMARK\_INTERFERON\_GAMMA\_RESPONSE | HALLMARK\_INTERFERON\_GAMMA\_RESPONSE | 52 | 1.2248309 | 0.11238095 | 0.5269397 |
| HALLMARK\_ANDROGEN\_RESPONSE | HALLMARK\_ANDROGEN\_RESPONSE | 28 | 1.2086315 | 0.21280992 | 0.51453006 |
| HALLMARK\_PI3K\_AKT\_MTOR\_SIGNALING | HALLMARK\_PI3K\_AKT\_MTOR\_SIGNALING | 19 | 1.1759912 | 0.096408315 | 0.53522223 |
| HALLMARK\_KRAS\_SIGNALING\_UP | HALLMARK\_KRAS\_SIGNALING\_UP | 39 | 1.1581504 | 0.1983471 | 0.57567394 |
| HALLMARK\_PEROXISOME | HALLMARK\_PEROXISOME | 23 | 1.1475459 | 0.21714285 | 0.5448098 |
| HALLMARK\_GLYCOLYSIS | HALLMARK\_GLYCOLYSIS | 40 | 1.0578392 | 0.40697673 | 0.766865 |
| HALLMARK\_INTERFERON\_ALPHA\_RESPONSE | HALLMARK\_INTERFERON\_ALPHA\_RESPONSE | 32 | 1.0567744 | 0.28761905 | 0.7121063 |
| HALLMARK\_BILE\_ACID\_METABOLISM | HALLMARK\_BILE\_ACID\_METABOLISM | 21 | 1.0415435 | 0.30285713 | 0.68508947 |
| HALLMARK\_ESTROGEN\_RESPONSE\_LATE | HALLMARK\_ESTROGEN\_RESPONSE\_LATE | 47 | 1.0257059 | 0.3946869 | 0.6725 |
| HALLMARK\_HEME\_METABOLISM | HALLMARK\_HEME\_METABOLISM | 33 | 1.0119822 | 0.41020793 | 0.659693 |
| HALLMARK\_MYOGENESIS | HALLMARK\_MYOGENESIS | 38 | 0.98338073 | 0.5009488 | 0.6764679 |
| HALLMARK\_ESTROGEN\_RESPONSE\_EARLY | HALLMARK\_ESTROGEN\_RESPONSE\_EARLY | 61 | 0.9683057 | 0.5984849 | 0.6753098 |
| HALLMARK\_MITOTIC\_SPINDLE | HALLMARK\_MITOTIC\_SPINDLE | 17 | 0.8800533 | 0.5168317 | 0.81293947 |
| HALLMARK\_ALLOGRAFT\_REJECTION | HALLMARK\_ALLOGRAFT\_REJECTION | 28 | 0.87049985 | 0.59090906 | 0.784934 |
| HALLMARK\_XENOBIOTIC\_METABOLISM | HALLMARK\_XENOBIOTIC\_METABOLISM | 29 | 0.8222649 | 0.601145 | 0.8242042 |
| HALLMARK\_KRAS\_SIGNALING\_DN | HALLMARK\_KRAS\_SIGNALING\_DN | 28 | 0.8191252 | 0.71428573 | 0.7976074 |
| HALLMARK\_P53\_PATHWAY | HALLMARK\_P53\_PATHWAY | 50 | 0.54662883 | 0.8952381 | 0.9702877 |
| NAME | SIZE | ES | NES | NOM p-val | FDR q-val | FWER p-val | RANK AT MAX | LEADING EDGE |
| --- | --- | --- | --- | --- | --- | --- | --- | --- |
| HALLMARK\_CHOLESTEROL\_HOMEOSTASIS | 32 | 0.46286732 | 1.2062927 | 0.17274472 | 1.0 | 0.559 | 544 | tags=53%, list=17%, signal=63% |
| HALLMARK\_UNFOLDED\_PROTEIN\_RESPONSE | 30 | 0.3903335 | 1.1198226 | 0.28709057 | 1.0 | 0.706 | 855 | tags=53%, list=26%, signal=72% |
| HALLMARK\_ADIPOGENESIS | 39 | 0.31163335 | 1.0617656 | 0.37037036 | 1.0 | 0.8 | 1156 | tags=62%, list=36%, signal=94% |
| HALLMARK\_ANDROGEN\_RESPONSE | 25 | 0.32563475 | 1.0422668 | 0.37672585 | 1.0 | 0.846 | 656 | tags=36%, list=20%, signal=45% |
| HALLMARK\_HYPOXIA | 53 | 0.27749223 | 1.0116172 | 0.265625 | 1.0 | 0.902 | 908 | tags=49%, list=28%, signal=67% |
| HALLMARK\_MTORC1\_SIGNALING | 70 | 0.45803115 | 1.0014694 | 0.4856046 | 1.0 | 0.902 | 671 | tags=53%, list=21%, signal=65% |
| HALLMARK\_GLYCOLYSIS | 44 | 0.41697046 | 0.9857369 | 0.3646833 | 1.0 | 0.902 | 1194 | tags=75%, list=37%, signal=117% |
| HALLMARK\_ESTROGEN\_RESPONSE\_LATE | 56 | 0.21684399 | 0.92393005 | 0.3696498 | 1.0 | 0.902 | 581 | tags=32%, list=18%, signal=38% |
| HALLMARK\_MITOTIC\_SPINDLE | 32 | 0.3561777 | 0.9173719 | 0.6026871 | 1.0 | 0.963 | 1498 | tags=81%, list=46%, signal=149% |
| HALLMARK\_APICAL\_JUNCTION | 45 | 0.26679942 | 0.88451374 | 0.4922179 | 1.0 | 0.963 | 1101 | tags=56%, list=34%, signal=83% |
| HALLMARK\_FATTY\_ACID\_METABOLISM | 29 | 0.22359753 | 0.8701712 | 0.4864341 | 1.0 | 0.963 | 414 | tags=24%, list=13%, signal=27% |
| HALLMARK\_E2F\_TARGETS | 35 | 0.35406044 | 0.8538261 | 0.6026871 | 1.0 | 0.963 | 1599 | tags=86%, list=49%, signal=167% |
| HALLMARK\_MYC\_TARGETS\_V1 | 18 | 0.34429264 | 0.8195928 | 0.45086706 | 1.0 | 0.963 | 1535 | tags=83%, list=47%, signal=157% |
| HALLMARK\_UV\_RESPONSE\_DN | 38 | 0.21789733 | 0.80997473 | 0.5029821 | 1.0 | 0.963 | 163 | tags=16%, list=5%, signal=16% |
| HALLMARK\_UV\_RESPONSE\_UP | 32 | 0.19322385 | 0.77962327 | 0.59108526 | 1.0 | 0.963 | 464 | tags=25%, list=14%, signal=29% |
| HALLMARK\_EPITHELIAL\_MESENCHYMAL\_TRANSITION | 77 | 0.16705617 | 0.74227524 | 0.7001972 | 1.0 | 0.963 | 671 | tags=30%, list=21%, signal=37% |
| HALLMARK\_MYOGENESIS | 39 | 0.19129561 | 0.73731303 | 0.701581 | 1.0 | 0.963 | 648 | tags=33%, list=20%, signal=41% |
| HALLMARK\_INFLAMMATORY\_RESPONSE | 43 | 0.17491712 | 0.7338405 | 0.57894737 | 1.0 | 0.963 | 513 | tags=26%, list=16%, signal=30% |
| HALLMARK\_G2M\_CHECKPOINT | 31 | 0.25468987 | 0.7312447 | 0.48737863 | 0.9589514 | 0.963 | 1599 | tags=77%, list=49%, signal=151% |
| HALLMARK\_IL2\_STAT5\_SIGNALING | 50 | 0.17263895 | 0.7236903 | 0.7889546 | 0.931403 | 0.963 | 787 | tags=36%, list=24%, signal=47% |
| HALLMARK\_BILE\_ACID\_METABOLISM | 22 | 0.18240984 | 0.69438237 | 0.8146718 | 0.92899394 | 0.963 | 656 | tags=32%, list=20%, signal=40% |
| HALLMARK\_DNA\_REPAIR | 22 | 0.16914035 | 0.6139541 | 0.70731705 | 0.98714656 | 0.963 | 2708 | tags=100%, list=83%, signal=590% |
| HALLMARK\_ESTROGEN\_RESPONSE\_EARLY | 59 | 0.14059617 | 0.55694133 | 0.8988327 | 1.0 | 1.0 | 762 | tags=31%, list=23%, signal=39% |
| HALLMARK\_PI3K\_AKT\_MTOR\_SIGNALING | 21 | 0.16152546 | 0.54058266 | 0.8974359 | 1.0 | 1.0 | 365 | tags=19%, list=11%, signal=21% |
| HALLMARK\_PEROXISOME | 18 | 0.13407758 | 0.5252761 | 0.8978389 | 0.9997693 | 1.0 | 1069 | tags=44%, list=33%, signal=66% |
| HALLMARK\_KRAS\_SIGNALING\_DN | 28 | 0.12607272 | 0.4077906 | 0.89980733 | 0.99815416 | 1.0 | 1725 | tags=71%, list=53%, signal=151% |
Supplemental Figure S2. KEGG Analysis of differentially-expressed genes upon DENSPM treatment in hPheo1 cells (A) and hPheo1_SDHB_kd cells (B).

## Slide 3
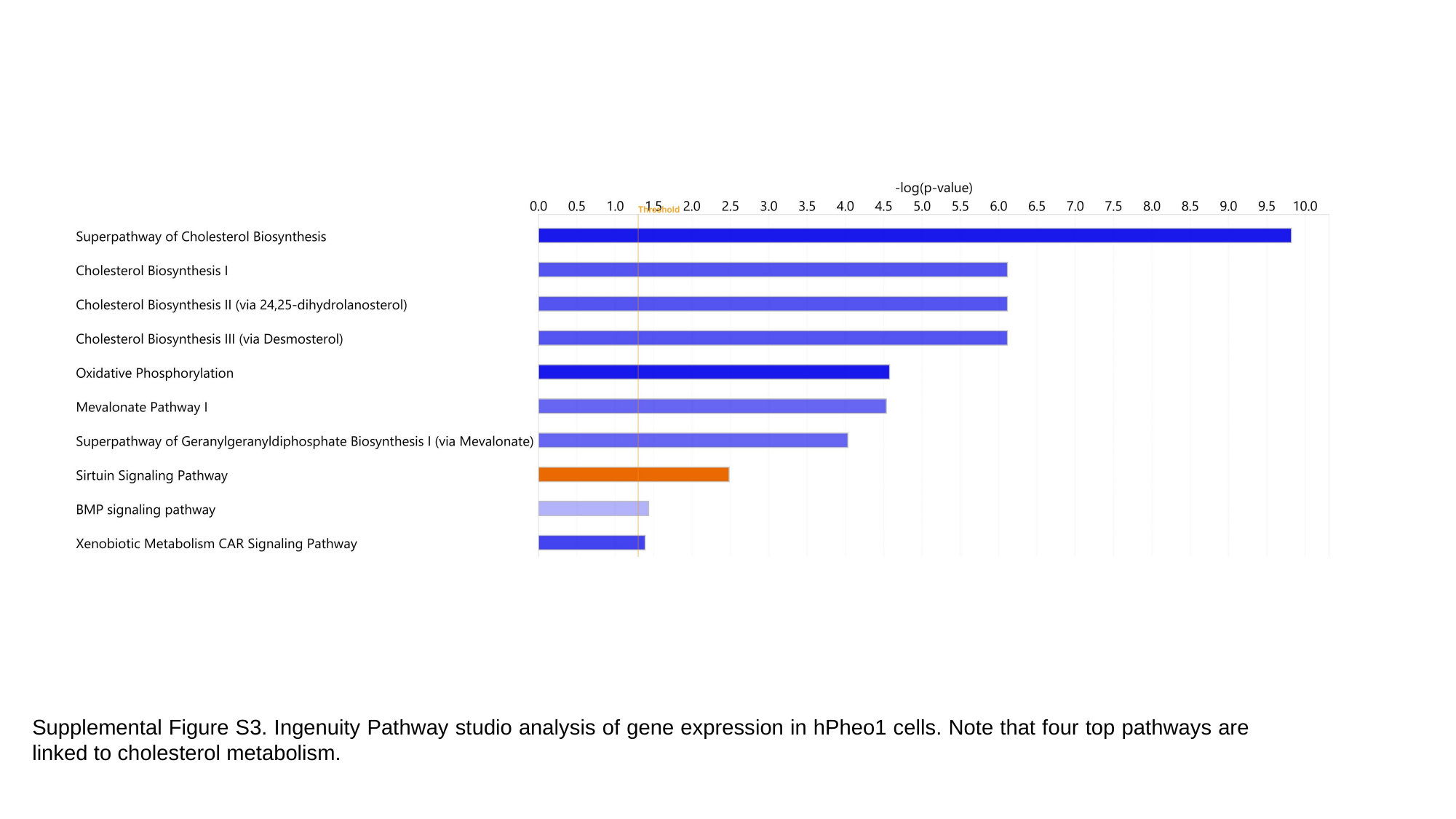

Supplemental Figure S3. Ingenuity Pathway studio analysis of gene expression in hPheo1 cells. Note that four top pathways are linked to cholesterol metabolism.

## Slide 4
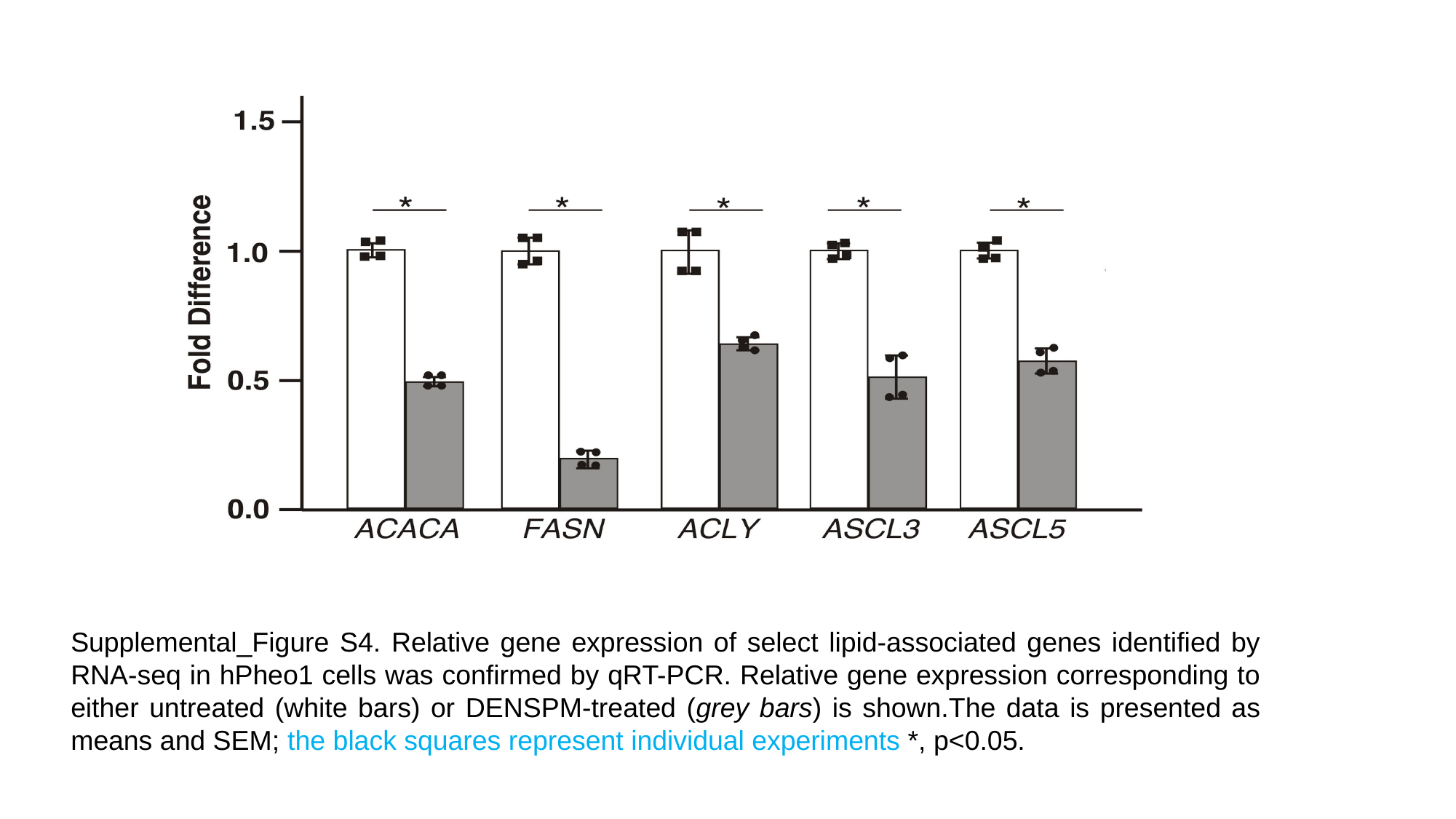

Supplemental_Figure S4. Relative gene expression of select lipid-associated genes identified by RNA-seq in hPheo1 cells was confirmed by qRT-PCR. Relative gene expression corresponding to either untreated (white bars) or DENSPM-treated (grey bars) is shown.The data is presented as means and SEM; the black squares represent individual experiments *, p<0.05.

## Slide 5
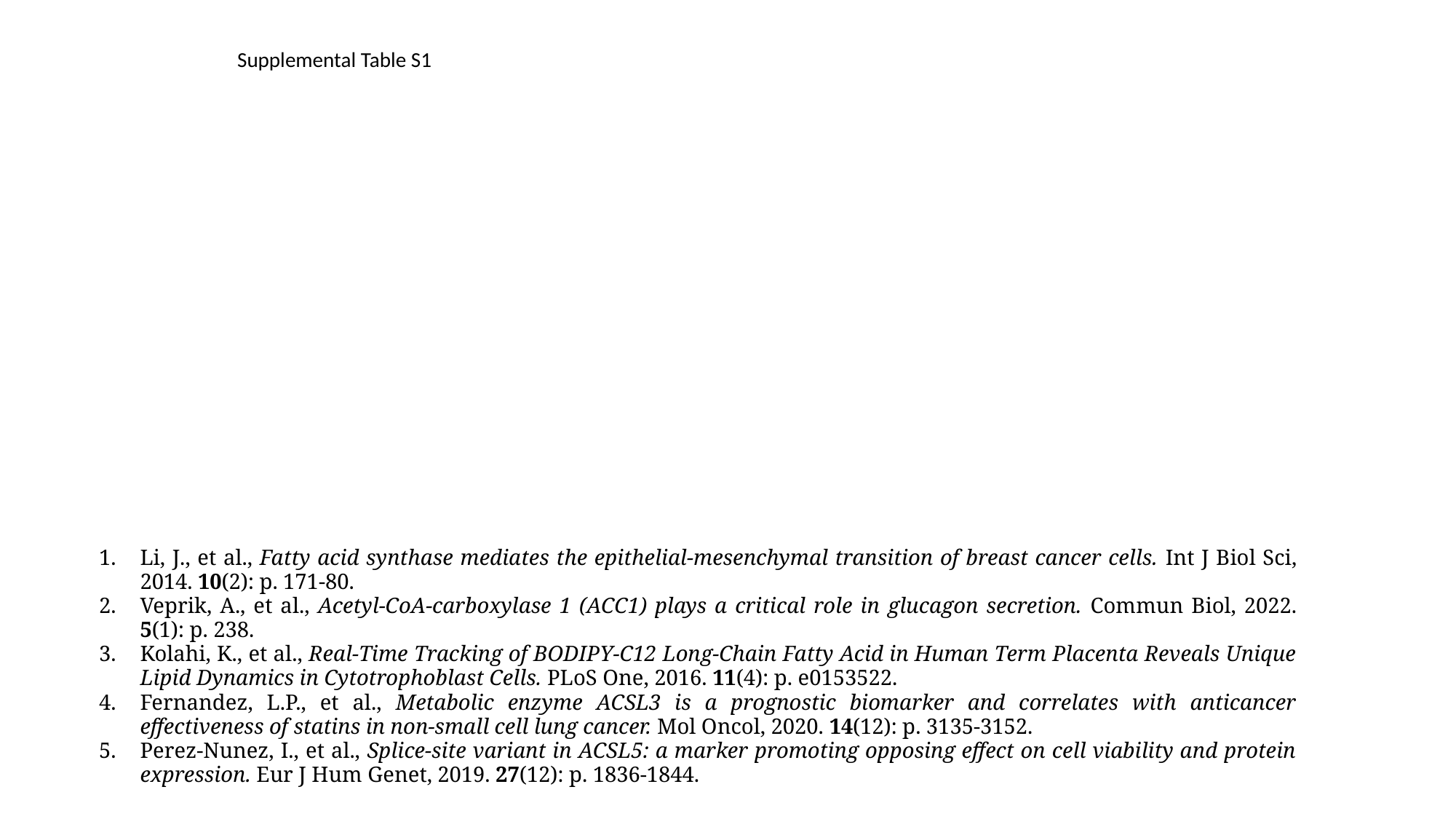

Supplemental Table S1
Li, J., et al., Fatty acid synthase mediates the epithelial-mesenchymal transition of breast cancer cells. Int J Biol Sci, 2014. 10(2): p. 171-80.
Veprik, A., et al., Acetyl-CoA-carboxylase 1 (ACC1) plays a critical role in glucagon secretion. Commun Biol, 2022. 5(1): p. 238.
Kolahi, K., et al., Real-Time Tracking of BODIPY-C12 Long-Chain Fatty Acid in Human Term Placenta Reveals Unique Lipid Dynamics in Cytotrophoblast Cells. PLoS One, 2016. 11(4): p. e0153522.
Fernandez, L.P., et al., Metabolic enzyme ACSL3 is a prognostic biomarker and correlates with anticancer effectiveness of statins in non-small cell lung cancer. Mol Oncol, 2020. 14(12): p. 3135-3152.
Perez-Nunez, I., et al., Splice-site variant in ACSL5: a marker promoting opposing effect on cell viability and protein expression. Eur J Hum Genet, 2019. 27(12): p. 1836-1844.
